# On the Role of Water in the Formation of a Deep Eutectic Solvent Based on NiCl_2_·6H_2_O and Urea

**DOI:** 10.1021/acs.inorgchem.2c00864

**Published:** 2022-05-26

**Authors:** Matteo Busato, Alessandro Tofoni, Giorgia Mannucci, Francesco Tavani, Alessandra Del Giudice, Andrea Colella, Mauro Giustini, Paola D’Angelo

**Affiliations:** Department of Chemistry, University of Rome “La Sapienza”, P.le A. Moro 5, Rome 00185, Italy

## Abstract

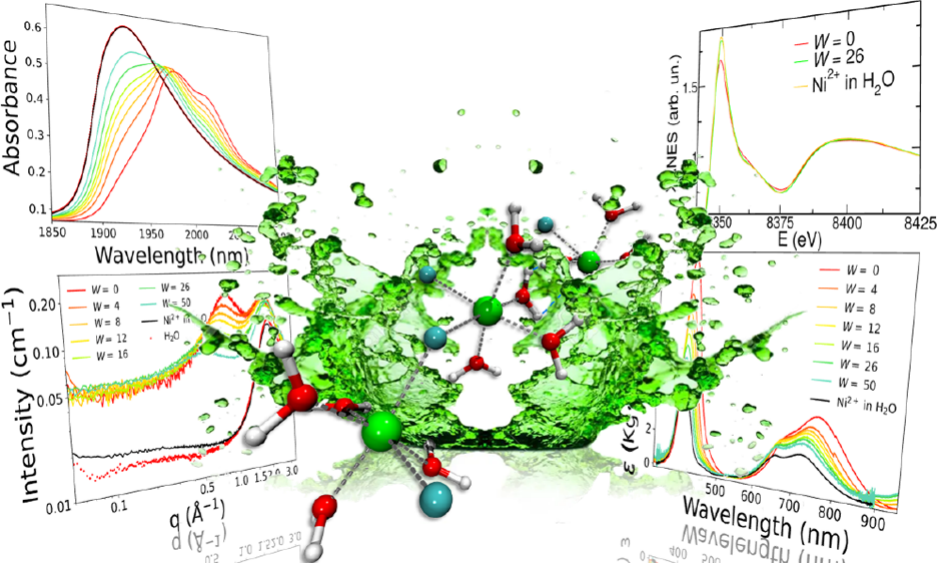

The metal-based deep eutectic solvent (MDES) formed by NiCl_2_·6H_2_O and urea in 1:3.5 molar ratio has been prepared for the first time and characterized from a structural point of view. Particular accent has been put on the role of water in the MDES formation, since the eutectic could not be obtained with the anhydrous form of the metal salt. To this end, mixtures at different water/MDES molar ratios (*W*) have been studied with a combined approach exploiting molecular dynamics and *ab initio* simulations, UV–vis and near-infra-red spectroscopies, small- and wide-angle X-ray scattering, and X-ray absorption spectroscopy measurements. In the pure MDES, a close packing of Ni^2+^ ion clusters forming oligomeric agglomerates is present thanks to the mediation of bridging chloride anions and water molecules. Conversely, urea poorly coordinates the metal ion and is mostly found in the interstitial regions among the Ni^2+^ ion oligomers. This nanostructure is disrupted upon the introduction of additional water, which enlarges the Ni–Ni distances and dilutes the system up to an aqueous solution of the MDES constituents. In the NiCl_2_·6H_2_O 1:3.5 MDES, the Ni^2+^ ion is coordinated on average by one chloride anion and five water molecules, while water easily saturates the metal solvation sphere to provide a hexa-aquo coordination for increasing *W* values. This multidisciplinary study allowed us to reconstruct the structural arrangement of the MDES and its aqueous mixtures on both short- and intermediate-scale levels, clarifying the fundamental role of water in the eutectic formation and challenging the definition at the base of these complex systems.

## Abstract 2

The metal-based deep eutectic solvent formed by NiCl_2_·6H_2_O and urea in 1:3.5 a molar ratio was prepared for the first time, and its aqueous mixtures were characterized from a structural point of view, highlighting the fundamental role of water in the eutectic formation.

## Introduction

The term “deep eutectic solvent” (DES) deals with a mixture formed by two or more compounds, which, at well-defined molar ratios, display a melting point (MP) that is not only lower than those of the single constituents but also lower than the ideally predicted one.^1^ In this way, a liquid phase can be obtained even from solid starting materials, as it was shown for the first time when Abbott and co-workers^2^ reported that by mixing choline chloride (ChCl) and urea, a eutectic with a MP of 12 °C was obtained for the 1:2 ratio, which was called “reline”. This behavior seems to rely on the extensive network of hydrogen-bonds (H-bonds) established among the components, which are often indicated as H-bond donor (HBD) and H-bond acceptor (HBA) species.^3−5^ The compositional heterogeneity of these solvents has led to the introduction of a classification based on the chemical nature of the constituents:^5^

Type I: quaternary ammonium + metal chloride salts (e.g., ChCl:ZnCl_2_);^6−9^

Type II: quaternary ammonium + metal chloride hydrate salts (e.g., ChCl:CrCl_3_·6H_2_O);^10,11^

Type III: quaternary ammonium salt + HBD (e.g., reline);^2^

Type IV: metal chloride salt + HBD (e.g., ZnCl_2_:urea).^12^

Despite the fact that three out of four of the proposed categories present a metal salt among the constituents, little attention has been paid so far to these kinds of eutectics, often indicated as “metal-based deep eutectic solvents” (MDESs), at least in comparison with the more studied “type III” ones.^5,13^ Nonetheless, many MDESs besides exhibiting intrinsic DES qualities such as easy preparation, no need for purification, and tunable physical–chemical properties have shown interesting properties addressing a wide horizon of technological applications.^6−11,14−18^ The high concentration of ionic species can indeed provide eutectic solvents with high polarity and conductivity, making these mixtures ideal candidates as new electrolytes and media for electrodepositions and catalytic processes. For example, the ChCl:CrCl_3_·6H_2_O 1:2 MDES was one of the first to be proposed, and it was demonstrated to provide black chromium deposits with excellent yields after electrodeposition, offering an alternative to Cr(VI) baths.^10,11^ Copper alloys and nanocrystalline nickel coatings have been deposited from MDESs formed by ChCl with CuCl_2_·2H_2_O and NiCl_2_·6H_2_O, respectively.^14,15^ On the other hand, the ChCl:ZnCl_2_ 1:2 MDES has been proposed both as a new electrolyte and as a catalytic environment promoting several organic reactions,^6−9^ while binary and tertiary mixtures of FeCl_3_, CoCl_2_, NiCl_2_, CuCl_2_, and ZnCl_2_ with HBAs like ChCl, ethylene glycol, and glycerol have been employed in the deep desulfurization of fuels and in the catalytic conversion of lignin.^16,17^ The MDES made by cerium nitrate and urea has been also observed to promote the self-assembly of different types of surfactants.^18^

It is remarkable that, in the above-mentioned applications, a controlled amount of water is often added to the MDES.^18−20^ The relationship between DESs and water is a long-standing debate, since water can be present in these eutectics up to high concentrations in both a desired and undesired way. The latter is often a consequence of the high hygroscopicity of many DESs, combined with the impossibility of operating in moisture-free conditions, while the former one offers a further design strategy to tailor DES physical–chemical properties. For example, the introduction of extra-water in ChCl:CrCl_3_·6H_2_O and ChCl:ZnCl_2_ has been found to reduce the MDES viscosity, while at the same time the water activity was low enough so that the electrochemical process could be operated at surprisingly high current efficiency.^19,20^ In addition, the quality of the deposited metallic alloy was found to be improved if water up to 20% w/w was added to the eutectic mixture. However, the impact of water on the DES structure and properties can provide a plethora of different situations depending on the specific system, leaving an amount of unanswered questions.^21−23^ This circumstance has the potential impact of undermining even the definition at the base of this class of solvents, as it was recently shown for the archetypal DES reline for which, under dry conditions, an MP of *ca*. 32 °C was obtained, which is much higher than the originally determined one.^24,25^ In the framework of MDES formation, some confusion in the present literature exists on the employment of either the anhydrous or the hydrated form of the metal salt, or on the control of the water uptake in the operating conditions.^6−9,26,27^ These issues, together with the well-known hygroscopicity of many metal chloride salts, induce suspicion about the effective hydration extent of the employed starting materials and on a possible involvement of water in the eutectic formation. In this framework, note that “type I” and “type II” DESs differ only with one being the hydrated form of the other.^5^

In this work, an MDES consisting of NiCl_2_·6H_2_O and urea in a 1:3.5 molar ratio is proposed for the first time, and we show that the eutectic cannot be obtained with the anhydrous form of the metal salt, *i.e.*, NiCl_2_. This MDES commensurate with the archetypal “type IV” ZnCl_2_:urea 1:3.5 presented by Abbott and co-workers,^12^ though an analogous CuCl_2_:urea 1:3.5 has been more recently proposed.^26^ In these works, it seems that the eutectic phase has been achieved with the anhydrous form of the metal salt, while very recently, various MDESs of lanthanide nitrates and urea still in the 1:3.5 molar ratio have been obtained with the hydrated form.^28^ In our opinion, this opens the question about the role played by water in the formation and in the structural arrangement of the MDES. To unveil this, mixtures at different water/MDES molar ratios (*W*) have been studied with a combined approach exploiting molecular dynamics (MD) and *ab initio* simulations, UV–vis and near-infra-red (NIR) spectroscopies, small- and wide-angle X-ray scattering (SWAXS), and X-ray absorption spectroscopy (XAS) measurements. The obtained multidisciplinary point of view allowed us to reconstruct the MDES structure on both short- and intermediate-scale levels. We believe that the considerations here reported offer a chance for the revaluation of the MDES nature and have the potential impact of challenging the DES classification in general.

## Materials and Methods

### Chemicals and Samples Preparation

NiCl_2_·6H_2_O (≥99%) and urea (≥99.5%) were purchased from Merck (Milan, Italy) and used as received. The NiCl_2_·6H_2_O:urea 1:3.5 MDES was prepared by mixing the components at the requested molar ratio in a glass test tube. The sample manipulation was carried out in an Ar-filled glove box (water content < 0.1 ppm) to prevent contact with the air moisture. The density of the NiCl_2_·6H_2_O MDES was calculated by weighing 1 mL of sample in a volumetric flask and resulted to be 1.506 g cm^–3^. MilliQ water was added to the MDES to prepare NiCl_2_·6H_2_O:urea:water 1:3.5:*W* mixtures with *W* = 4, 8, 12, 16, 26, and 50.

### MD Simulations

Classical MD simulations were performed on NiCl_2_·6H_2_O:urea:water systems at different 1:3.5:*W* molar ratios. Cubic boxes were built with ∼50 Å side lengths by randomizing the atomic positions with the PACKMOL package.^29^ For the pure MDES, the number of species was chosen to reproduce the experimental density, while for the aqueous mixtures it reproduces the density calculated by assuming ideality from the molar compositions and the molecular weights (*MW*) of the components with the following equation:
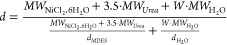
1where *d_MDES_* and *d*_H_2_O_ are the MDES and water experimental densities at 25 °C, respectively. This strategy has been previously employed for other DES mixtures with cosolvents,^21,23^ while its applicability on the present system has been assessed by comparing the computed density with the experimental one for selected samples. As a result, they were found to diverge less than 5%. The details of the simulated systems are listed in Table S1. The structures and interactions of the urea molecule and of the chloride anion were represented by the OPLS/AA force field,^30^ while the SPC/E model was employed for water. The Lennard–Jones (LJ) parameters for the Ni^2+^ ion were taken from the “IOD set” by Li *et al*.^31^ The cross-terms for the LJ interactions were constructed with the Lorentz–Berthelot combining rules. The charges of the ionic species were scaled by a factor of 0.9, as this strategy was previously demonstrated to improve the transport properties and to take into account polarization effects implicitly.^32−35^ A cutoff radius of 12 Å was employed for all the non-bonded interactions, while the long-range electrostatic forces were computed with the particle mesh Ewald method.^36,37^ All the stretching vibrations involving the hydrogen atoms were constrained with the LINCS algorithm.^38^ The employed simulation protocol is reported elsewhere,^4,39−43^ though details are provided in the SI.

### SWAXS Measurements

The X-ray scattering measurements were carried out at the SAXSLab Sapienza with a Xeuss 2.0 Q-Xoom system (Xenocs SAS, Grenoble, France), equipped with a micro-focus Genix 3D X-ray source (λ = 1.542 Å), a two-dimensional Pilatus3 R 300 K detector that can be placed at variable distances from the sample, and an additional Pilatus 100 K detector at a fixed position to access wider angles (Dectris Ltd., Baden, Switzerland). The calibration of the scattering vector *q* range, where *q* = (4πsinθ)/λ and 2θ is the scattering angle, was performed with a silver behenate standard for the SAXS detector and Al_2_O_3_ for the WAXS one. The beam size was defined through the two-pinhole collimation system equipped with “scatterless” slits to be 0.5 mm × 0.5 mm. Measurements with a sample-detector distance of 26.2 cm were performed, and the overall explored *q* region was 0.05–3.26 Å^–1^. The NiCl_2_·6H_2_O:urea:water mixtures at different 1:3.5:*W* molar ratios and, as references, a 50 mM NiCl_2_ aqueous solution and pure water were loaded into vacuum-tight cells with flexible Kapton windows and a steel spacer with a nominal thickness of 0.05 cm and placed within the holder in the sample chamber at reduced pressure (∼0.2 mbar). Measurements were carried out at room temperature. Details about the data reduction are given in the SI.

### UV–vis and NIR Measurements

Absorption spectra were recorded in the UV–vis and NIR regions at room temperature on the NiCl_2_·6H_2_O:urea:water mixtures at different 1:3.5:*W* molar ratios. The spectra were collected also on a 50 mM NiCl_2_ aqueous solution, on pure water, and on a solid pellet of 10% w/w urea in KBr, as references. The measurements were carried out with a Varian Cary 5E UV–vis–NIR spectrometer using a quartz cell with 0.01 cm optical length. Absorbances were measured with an integration time of 0.1 s and a 0.5 nm interval over the 250–1000 and 1850–2100 nm range for the UV–vis and NIR regions, respectively. The raw UV–vis spectra were baseline subtracted using the SpectraGryph program^44^ employing a linear background and were presented as the molal absorption coefficient of the Ni^2+^ ion *vs* wavelength, due to the high viscosity of the samples preventing the measurement of their volumes with the sufficient degree of accuracy. The values of the Ni^2+^ ion molality for the different samples are listed in Table S2.

### *Ab Initio* Calculations

The electronic transitions were simulated from *ab initio* calculations at the complete active space self consistent field (CASSCF) level of theory supported by the strongly correlated N-electron valence state perturbation theory (SC-NEVPT2)^45^ for octahedral clusters with different Ni^2+^ coordinations, namely [Ni(H_2_O)_6_]^2+^, [NiCl(H_2_O)_5_]^+^, [NiCl_2_(H_2_O)_4_] (*cis*), [NiCl_2_(H_2_O)_4_] (*trans*), [NiCl_3_(H_2_O)_3_]^−^, and [NiCl_4_(H_2_O)_2_]^2–^. To this purpose, gas phase CASSFCF/NEVPT2 calculations were performed with the (8,5) and (14,8) active spaces on the geometries optimized at the density functional theory (DFT) level both in the gas phase and in the presence of water introduced as implicit solvent with the PCM model.^46^ More details about the employed simulation protocol and level of theory are reported in the SI.

### X-ray Absorption Spectroscopy

Ni K-edge XAS spectra were collected on the NiCl_2_·6H_2_O:urea:water 1:3.5:*W* mixtures with *W* = 0 (pure MDES) and 26 at the 11.1 beamline^47^ of Elettra-Sincrotrone Trieste (Italy) in transmission geometry. Owing to the high metal concentration of the samples, a precise amount of each mixture was laid on a cellulose membrane, which was then sealed on both sides with a Mylar tape. Measurements were carried out with a Si(111) double crystal monochromator, while the storage ring was operating at 2 GeV and the beam current was 200 mA. At least three spectra were recorded and averaged for each sample. XAS data were also collected on a 0.2 M Ni(NO_3_)_2_ solution in water as a comparative system. The experimental setup for this sample can be found elsewhere.^48−51^ The analysis of the EXAFS and XANES part of the collected spectra was carried out with the procedures reported in the SI.

## Results and Discussion

As stated above, the main effort of this work is to get insights on the structural arrangement of the NiCl_2_·6H_2_O:urea 1:3.5 MDES and its aqueous mixtures, with the aim of understanding the role of water in the eutectic formation. In the following section, we initially report the experimental observations obtained from the MDES preparation; then, the MD results are discussed as this method is ideally suited to provide an overview on the structural aspects of the studied systems ranging from short- to long-range lengths. Subsequently, the SWAXS and NIR outcomes are presented to confirm and deepen the observed intermediate-range structure, while the UV–vis absorption and the analysis of the XAS data are intended to probe the local environment around the Ni^2+^ ion. In this way, the whole results are exposed as a digression from larger to shorter scale lengths, allowing the achievement of an all-round picture of the MDES structural aspects.

### MDES Formation

**Figure 1:**
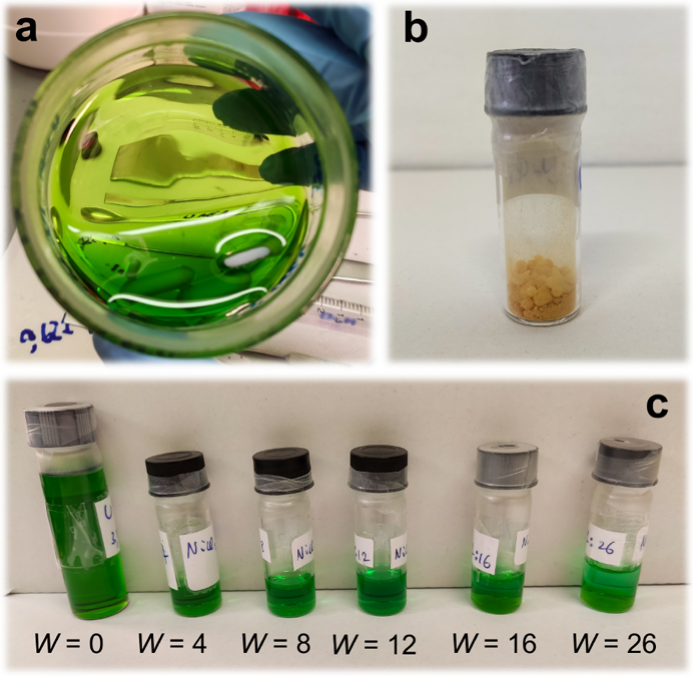
Photographs of (a) the NiCl_2_·6H_2_O:urea 1:3.5 MDES, (b) NiCl_2_ and urea reagents mixed in a 1:3.5 molar ratio, and (c) some of the prepared NiCl_2_·6H_2_O:urea:water mixtures at different 1:3.5:*W* molar ratios.

The eutectic was found to occur at a NiCl_2_·6H_2_O:urea molar ratio of 1:3.5. At this composition, it is a homogeneous and bright green liquid with high viscosity (Figure 1a). The MDES was observed to form overnight at room temperature under mere magnetic stirring. Heating was avoided also because the thermal decomposition of urea is believed to cause the precipitation of metal carbonates, as previously observed for similar MDESs based on urea and lanthanide nitrates.^28^ Any attempt to obtain the eutectic with the anhydrous form of the metal salt, *i.e.*, NiCl_2_:urea 1:3.5, did not provide a liquid mixture even after 9 months (Figure 1b). This is a first suggestion that the hydration water molecules have a vital role in the eutectic formation.

The dilution of the MDES with additional water to obtain the NiCl_2_·6H_2_O:urea:water mixtures at different 1:3.5:*W* molar ratios did not bring to an appreciable variation of the color of the solutions, at least by the naked eye, even if this effect was expected to occur due to a possible change in the metal ion coordination (Figure 1c). Note that the formation of a milky precipitate has been in any case observed in the aqueous mixtures of the MDES after sample storage for a long time. We believe that this is more likely connected with the hydrolysis promoted by the urea basicity, as previously reported for metal salt solutions in the reline DES.^52^ A suitable strategy to circumvent this problem would consist of the employment of a low concentrated (*e.g.*, 0.01 M) acidic solution instead of pure water, as previously suggested.^53−56^

### Overview of the MDES Structural Arrangement: MD Results

**Figure 2:**
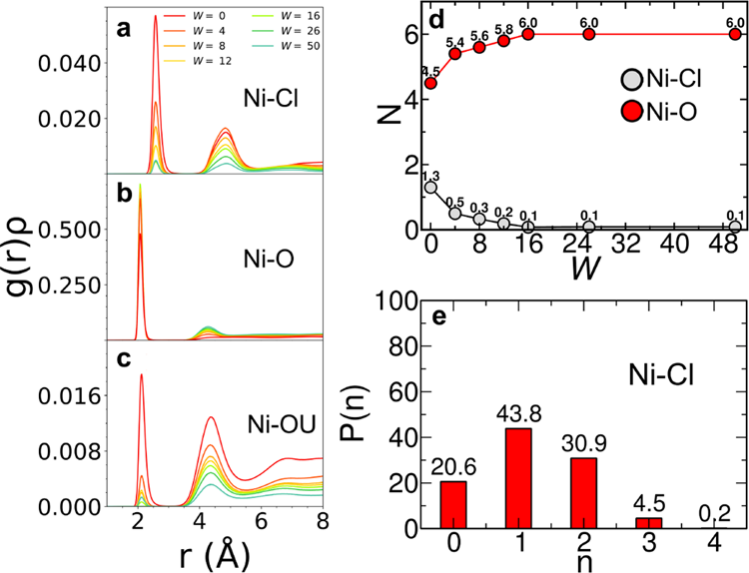
Radial distribution functions multiplied by the numerical densities of the observed atoms, *g*(*r*)ρ’s, calculated from the MD simulations of the NiCl_2_·6H_2_O:urea:water mixtures at different 1:3.5:*W* molar ratios between the Ni^2+^ ion and (a) the chloride anion and the oxygen atom of the (b) water and (c) urea molecules. (d) Corresponding coordination numbers *N*, taken at the first minimum of the *g*(*r*)ρ’s, for the Ni–Cl and Ni–O–pairs, plotted as a function of *W*. (e) Instantaneous coordination number *n* distribution, expressed in percentage, for the Ni–Cl pair in the *W* = 0 system.

A description of the local structure around the Ni^2+^ ion has been achieved by calculating the radial distribution functions *g*(*r*)’s for the Ni–Cl, Ni–O, and Ni–OU pairs with the chloride anion and with the oxygen atoms of the water and urea molecules, respectively, from the MD simulations of the NiCl_2_·6H_2_O:urea:water systems at different 1:3.5:*W* molar ratios (Figure 2a–c). In the NiCl_2_·6H_2_O:urea 1:3.5 MDES (*W* = 0), an average number of 1.3 chloride anions and 4.5 water molecules are found in the first solvation sphere of the metal ion, as shown by the obtained coordination number *N* values (Figure 2d). On the other hand, the Ni–OU distribution shows a lower intensity, integrating only 0.3 for *W* = 0 (Figure 2c). This evidence suggests that urea poorly coordinates the Ni^2+^ ion, which in turn forms octahedral complexes almost only with the chloride anions and the water molecules. Nonetheless, the fractional *N* values obtained subtend a more complicated composition of the metal solvation sphere. To unveil this behavior, the distribution of the instantaneous Ni–Cl coordination number *n* across the MD trajectory has been computed and is shown in Figure 2e. As a result, even if the Ni^2+^ ions are coordinated by one chloride anion in 43.8% of the cases, the two-fold Cl^–^ arrangement is also remarkable (30.9%) and at the same is true for the case in which the chloride anions do not coordinate the metal (20.6%). The combined distribution function (CDF) between the Ni–Cl distances and the Cl–Ni–Cl angles has been also calculated, and two spots of high intensity at 90 and 180° are observed (Figure S1), showing that the chloride anions can coordinate the metal both in *cis* and *trans* configurations. The whole evidence suggests that the Ni^2+^ ion speciation in the NiCl_2_·6H_2_O:urea 1:3.5 MDES is more realistically described by the coexistence of the [Ni(H_2_O)_6_]^2+^, [NiCl(H_2_O)_5_]^+^, and [NiCl_2_(H_2_O)_4_] species, rather than by the establishment of a single defined complex. Once water is added to the MDES, the intensity of the Ni–O *g*(*r*) is found to increase, while the Ni–Cl one decreases, as expected (Figure 2a,b, respectively). This behavior is reflected by the evolution of the corresponding *N* values (Figure 2d), showing that the hexa-aquo coordination is easily obtained once water is added to the MDES.

**Figure 3:**
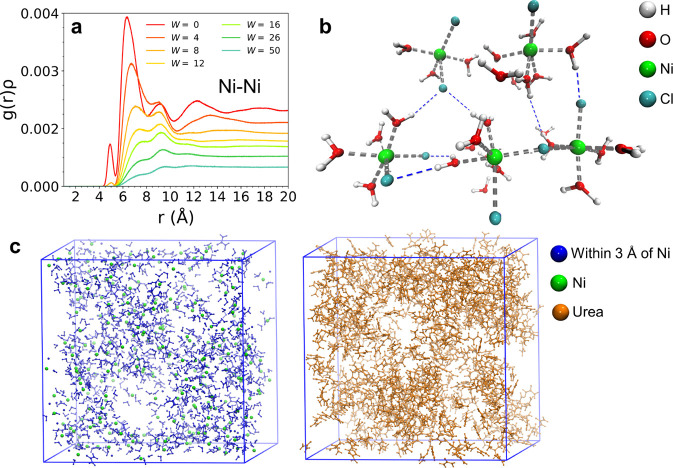
(a) Radial distribution functions multiplied by the numerical densities of the observed atoms, *g*(*r*)*ρ*’s, calculated for the Ni–Ni pair from the MD simulations of the NiCl_2_·6H_2_O:urea:water mixtures at different 1:3.5:*W* molar ratios. (b) Selected MD snapshot showcasing the close contacts between the Ni^2+^ ion clusters in the NiCl_2_·6H_2_O:urea 1:3.5 MDES (gray dashed lines: coordination interactions, blue dashed lines: H-bonds). (c) Snapshot taken from the final MD configuration of the NiCl_2_·6H_2_O:urea 1:3.5 MDES (left panel: species within 3 Å of the Ni^2+^ ions, right panel: urea). The different species are shown according to the reported color-code.

Information about the relative spatial arrangement of the above-mentioned Ni^2+^ ion clusters can be gained from the Ni–Ni *g*(*r*)’s reported in Figure 3a. In the NiCl_2_·6H_2_O:urea 1:3.5 MDES, this distribution shows an intense peak with a maximum at 6.37 Å. This is a remarkably short-range distance for 2+ charged ions and suggests that oligomeric agglomerates of Ni^2+^ complexes are formed in the eutectic. Such a closed packing seems to be possible thanks to the mediation of the interstitial chloride anions and water molecules, as shown by the representative snapshot in Figure 3b. Here, it can be observed that the chloride anions are able to bridge between different Ni^2+^ centers and that the coordinating water molecules form H-bonds with other ligands that in turn coordinate a further metal ion. Note that this structural arrangement is promoted by the versatile role of the water molecules that are able to act both as HBD and HBA, clarifying their importance in the MDES formation and explaining the impossibility to obtain the eutectic mixture with the anhydrous form of the metal salt (Figure 1b). Integration of the Ni–Ni *g*(*r*) at the first minimum after the main peak provides a value of 4.6 for *W* = 0. Being Ni^2+^ hexa-coordinated, this value suggests that, among the six ligands interacting with each Ni^2+^ ion, about 4–5 of them are able to bridge with another solvation complex. The whole picture is therefore evocative of a plethora of interactions that are established in the interstitial regions among the metal clusters to keep this oligomeric structure together. Moving to a larger distance scale, the snapshot taken on the entire simulation box reported in Figure 3c reveals that the Ni^2+^ ion clusters are somehow segregated from the urea, which is interspersed among these regions rich in Ni^2+^, water, and Cl^–^. This is consistent with the negligible intensity of the Ni-OU *g*(*r*) (Figure 2c), suggesting that the marginal Ni–urea interactions probably interest only the peripheral regions occupied by the oligomeric Ni^2+^ ion clusters. In this way, a secondary picture of the MDES nanostruscure becomes evident and suggests also the role of the urea, that is, of acting as a sort of “inner solvent” and lubricating the Ni-rich regions. As it is known, the properties of binary DESs are often interpreted in light of the interplay between the two precursors.^3−5^ In this framework, the key to understand the nature of the NiCl_2_·6H_2_O:urea 1:3.5 MDES seems to pass through the identification of the urea molecules and of the hydrated/chlorinated Ni^2+^ clusters, rather than of the bare Ni^2+^ ions, as the main partners of the eutectic formation, since the metal centers are totally immersed in an environment made by water molecules and chloride anions forming the previously mentioned oligomeric network. It is noteworthy to observe that the composition of the Ni^2+^ ion coordination sphere closely resembles that of the NiCl_2_·6H_2_O crystal, which is found to be substantially preserved even after the eutectic formation. The effect on this structure provoked by the introduction of additional water can be drawn from the evolution of the Ni–Ni *g*(*r*)’s for increasing *W* values (Figure 3a), showing that this distribution becomes broader and shifts at longer distances. This suggests that, once the Ni^2+^ coordination sphere is saturated, the water molecules tend to fill the regions of space among the metal clusters and separate them from each other until the dilution of the system resembles more and more a solution of NiCl_2_ and urea in water.

### Intermediate-Range Structure and Interstitiality: SWAXS and NIR Results

**Figure 4:**
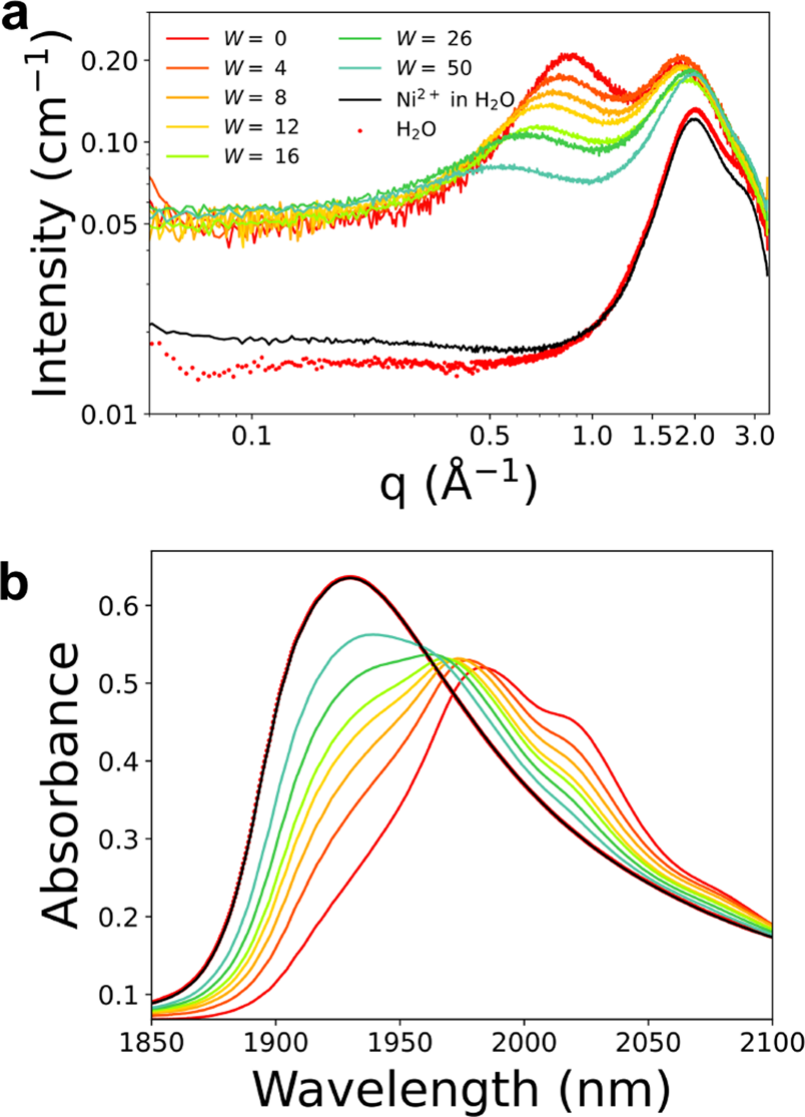
Log–log plot of the experimental SWAXS data (a) and NIR absorption spectra (b) for the NiCl**_2_·**6H_**2**_O:urea:water mixtures at different 1:3.5:*W* molar ratios, for a 50 mM NiCl_2_ aqueous solution, and for pure water.

To have an experimental confirmation of the intermediate-range structures observed by the MD simulations, SWAXS data have been collected on the NiCl_2_·6H_2_O:urea:water mixtures at different 1:3.5:*W* molar ratios. The scattering profiles are shown in Figure 4a and compared to those obtained for a 50 mM NiCl_2_ aqueous solution and for pure water, as references. All the spectra of the mixtures display a characteristic feature, *i.e.*, the appearance of a prepeak in the wide-angle X-ray scattering (WAXS) region. Such a contribution is known to be related to the presence of electron density inhomogeneities at recurring distances.^4,21,23,57^ In the NiCl_2_·6H_2_O:urea 1:3.5 MDES, the maximum of this peak is placed at a *q* value of ∼0.85 Å^–1^, corresponding to a distance of ∼7.3 Å in the real space. This value is consistent with the position of the maximum in the Ni–Ni *g*(*r*) for *W* = 0 (Figure 2a), strongly suggesting that this feature arises from the Ni–Ni distances as a result of the oligomeric network formed by the Ni^2+^ ion clusters. Water addition to the MDES provokes both a broadening and a shift of this prepeak to lower *q* values (Figure 4a) and thus to longer distances in the direct space. This trend nicely correlates with the evolution of the Ni–Ni *g*(*r*)’s (Figure 3a), which also become wider and shifted toward longer distances upon water addition. With regards to the segregation between the urea and the Ni-rich regions suggested by the MD results, the related larger-scale electron density heterogeneities should result in an increase of the small-angle signal intensity.^21^ The absence of such a clear feature in the experimental scattering profiles (Figure 4a) could be indicative of the transient nature of these structures rather than the establishment of individual mesophase domains with long lifetimes, so that the MDES results in an average homogeneity in terms of electron density when seen at the tens of Å scale. We also observe that the scattered intensity in the low-*q* region shows much higher values if compared with the reference samples such as the 50 mM NiCl_2_ aqueous solution and pure water (Figure 4a). This arises from the massive presence of electron-rich centers in the NiCl_2_·6H_2_O:urea 1:3.5 MDES, with large atomic X-ray scattering factors and inter-atomic correlation contributions, provided by the high Ni^2+^ and chloride concentrations. As a result, small contributions from the above-mentioned possible fluctuations would be hard to detect.

The speciation of water in the MDES and in its aqueous mixtures was better characterized from the water absorption in the NIR region. In this spectral range, water is known to have a diagnostic combination band due to the mixing of scissoring and asymmetric stretching vibrations, which has been extensively employed to characterize several aqueous systems.^58−60^Figure 4b collects the NIR spectra of the NiCl_2_·6H_2_O:urea:water mixtures at different 1:3.5:*W* molar ratios. As can be observed, the spectral profile for *W* = 0 shows the presence of different contributions. In particular, the peak at *ca*. 1980 nm and the two shoulders at 2020 and 2080 nm can be attributed to the urea molecule, as confirmed by the NIR spectrum collected on the solid compound (Figure S2). These bands are found to decrease in intensity and show a blue-shift upon water addition (Figure 4b), probably as a combination of the system dilution and of a change in the urea interactions. Conversely, the spectral feature at lower wavelengths is diagnostic for the water contribution. This band appears as a shoulder for *W* = 0, while it intensifies and blue-shifts at increasing *W* values, up to the characteristic ∼1930 nm band of bulk water.^61^ The blue-shift could be related with the fact that, in the NiCl_2_·6H_2_O:urea 1:3.5 MDES, almost the whole amount of the water contained in the sample is coordinated to the Ni^2+^ centers, and this is translated in a lower energy of the vibration modes contributing in this region. Upon increasing *W* values, water saturates the Ni^2+^ ions and tends to fill the regions of space among the metal clusters, behaving more and more as bulk water, in agreement with the picture obtained by the MD simulations.

### Determination of Ni^2+^ Speciation by UV–vis Absorption

**Figure 5:**
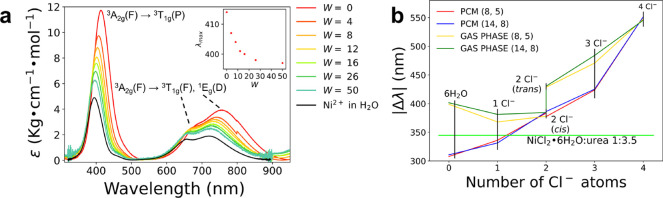
(a) UV–vis absorption spectra of the NiCl_2_·6H_2_O:urea:water mixtures at different 1:3.5:*W* molar ratios and of a 50 mM NiCl_2_ aqueous solution. The maxima positions λ_*MAX*_ of the ^3^A_2*g*_(F) → ^3^T_1*g*_(P) transition band are reported in the inset as a function of *W*. (b) Differences Δλ between the positions of the maxima of the two most intense transitions in the 350–450 and 600–1050 nm regions computed at the CASSCF/NEVPT2 level with the (8,5) and (14,8) active spaces, reported in function of the number of chloride anions in the clusters optimized both in the gas phase and in PCM water. Horizontal green line: value obtained for the difference between the maxima of the ^3^A_2*g*_(F) → ^3^T_1*g*_(P) and ^3^A_2*g*_(F) → ^3^T_1*g*_(F),^1^E_*g*_(D) transitions for the experimental UV–vis spectrum of the NiCl_2_·6H_2_O:urea 1:3.5 MDES.

The presence of partially filled *d*-orbitals in the Ni^2+^ ion provides useful spectroscopic handle of its compounds, offering a suitable strategy for the determination of its speciation in solution. The high sensitivity of the UV–vis spectroscopy to the changes in the local environment of the absorbing center has been therefore exploited to get more accurate information on the NiCl_2_·6H_2_O:urea 1:3.5 MDES and on its aqueous mixtures. The recorded absorption spectra are shown in Figure 5a together with those collected on a 50 mM NiCl_2_ aqueous solution as reference. The obtained spectral profiles show the typical fingerprint of the Ni^2+^ ion in an octahedral field across the entire composition range, as previously observed in a wide variety of solvent conditions.^54−56,62−64^ Octahedral Ni^2+^ complexes are known to provide three major spin-allowed transitions from the ground state: ^3^A_2*g*_(F) → ^3^T_2*g*_(F), ^3^T_1*g*_(F), ^3^T_1*g*_(P).^54,62^ The ^3^A_2*g*_(F) → ^3^T_2*g*_(F) transition lies in the IR region and is therefore outside the explored spectral range. The peaks located between *ca*. 400 and 430 nm correspond to the ^3^A_2*g*_(F) → ^3^T_1*g*_(P) one. The double bands between 600 and 900 nm are related to the ^3^A_2*g*_(F) → ^3^T_1*g*_(F) plus a shoulder corresponding to the ^3^A_2*g*_(F) →^1^E_*g*_(D) forbidden transition, due to the spin–orbit coupling mixing the ^3^T_1*g*_(F) and ^1^E_*g*_(D) states.^54,62^ The positions of the peak maxima for these transitions are listed in Table S3. Note that the absorption intensities connected to the octahedral coordination are orders of magnitude lower than those found in the tetrahedral case, due to the presence of an inversion center in the octahedral geometry.^62^ The formation of even little tetrahedral coordination in solution would be therefore easily detectable due to the appearence of the well-known double band between 600 and 800 nm corresponding to the ^3^T_1*g*_(F), ^3^T_1*g*_(P) transition. The absence of this spectral feature can be therefore taken as a direct proof of the fully octahedral coordination of the Ni^2+^ ion in the NiCl_2_·6H_2_O:urea 1:3.5 MDES and in its aqueous mixtures. This circumstance is typical of the Ni^2+^ ion, since in aqueous solutions with increasing chloride concentration, the octahedral coordination has been previously observed to predominate within the whole explored salinity range, while the transition to the tetrahedral one has occurred only for high temperatures and pressures.^56,54^ Once water is added to the NiCl_2_·6H_2_O:urea 1:3.5 MDES, a systematic shift of the transition maxima at higher energies can be observed for all the three bands (Figure 5a). This blue-shift is consistent with a gradual replacement of the chloride anions by the water molecules, the latter one being a stronger ligand than the former. The whole result therefore confirms that, in the NiCl_2_·6H_2_O:urea 1:3.5 MDES, the Ni^2+^ ion is not fully coordinated by water, but a certain amount of chloride anions is able to enter the metal solvation sphere. Note that the high sensitivity of the UV–vis technique allowed us to detect a physical observable that could not be observed as a color shift of the samples upon water addition (Figure 1c).

To obtain a more quantitative insight into the number of chloride anions coordinating the Ni^2+^ ion in the NiCl_2_·6H_2_O:urea 1:3.5 MDES, the electronic transitions have been simulated by means of *ab initio* calculations for octahedral clusters with an increasing number of Cl^–^ anions, namely [Ni(H_2_O)_6_]^2+^, [NiCl(H_2_O)_5_]^+^, [NiCl_2_(H_2_O)_4_] (*cis*), [NiCl_2_(H_2_O)_4_] (*trans*), [NiCl_3_(H_2_O)_3_]^−^, and [NiCl_4_(H_2_O)_2_]^2–^. The optimized structures are shown in Figure S3, while selected geometric parameters are listed in Table S4. All the simulated bands are shown in Figure S4, while in Figure 5b, we report the differences Δλ between the positions of the maxima of the two most intense transitions in the 350–450 and 600–1050 nm regions computed at the CASSCF/NEVPT2 level in the gas phase with both the (8,5) and (14,8) active spaces as a function of the number of coordinating chloride anions for the clusters optimized both in the gas phase and in PCM water. Table S5 lists the vertical transition energies and the oscillator strengths obtained for these transitions. This result is compared with the value obtained for the difference between the maxima of the ^3^A_2*g*_(F) → ^3^T_1*g*_(P) and ^3^A_2*g*_(F) → ^3^T_1*g*_(F),^1^E_*g*_(D) transitions for the experimental UV–vis spectrum of the NiCl_2_·6H_2_O:urea 1:3.5 MDES (green line in Figure 5b). As can be observed, the best match with the experimental Δλ value is provided by the [NiCl(H_2_O)_5_]^+^ cluster, while the increase of the number of coordinating Cl^–^ anions results in a progressive displacement from the experimental determination. This result suggests that the preferential coordination of the Ni^2+^ ions in the MDES involves on average one chloride anion and five water molecules, this being a first confirmation of the average Ni^2+^ speciation obtained from the MD simulations (Figure 2).

### Determination of Ni^2+^ Speciation by XAS

**Figure 6:**
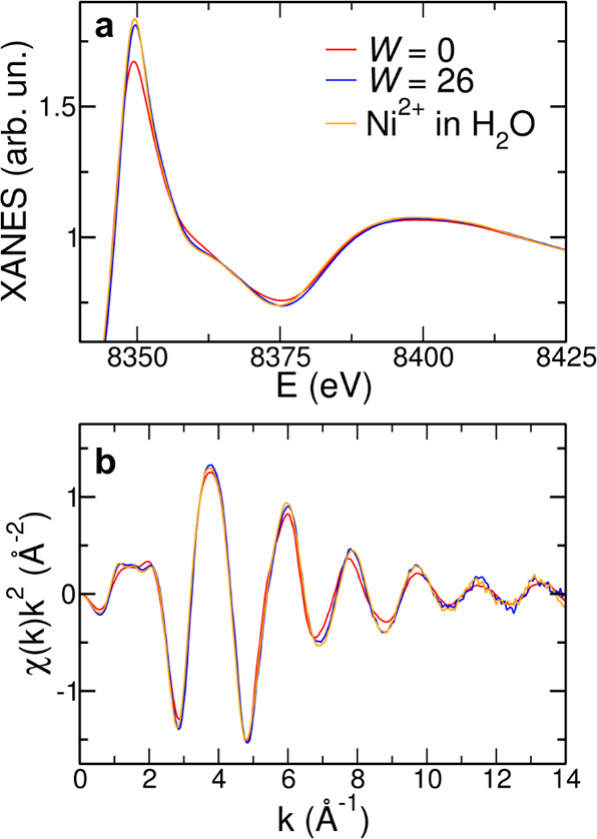
Ni K-edge (a) normalized XANES and (b) EXAFS experimental spectra collected on the NiCl_2_·6H_2_O:urea:water 1:3.5:*W* samples with *W* = 0 and 26 compared to the absorption spectra of a 0.2 M Ni(NO_3_)_2_ aqueous solution.

When dealing with metal ion solutions, XAS is the technique of choice to obtain an accurate determination of the local structure around the photoabsorbing center.^39−42,65,66^ XAS spectra have been collected on the NiCl_2_·6H_2_O:urea 1:3.5 MDES and on the NiCl_2_·6H_2_O:urea:water 1:3.5:26 mixture, and they are shown in Figure 6. Here, the data are compared to the XAS spectrum collected on a 0.2 M Ni(NO_3_)_2_ aqueous solution as the reference system. A first qualitative information can be obtained from the XANES region (Figure 6a), which is known to be dominated by the MS effects and is thus very sensitive to the three-dimensional distribution of the scattering atoms around the photoabsorber.^65,66^ From the normalized XANES data, it can be observed that the spectrum of the NiCl_2_·6H_2_O:urea 1:3.5 MDES differs from that of the *W* = 26 mixture and of the Ni(NO_3_)_2_ aqueous solution. In particular, in the XANES spectra of Ni^2+^ in water and in the *W* = 26 mixture, there is a bump at about 8360 eV that is smeared out in the *W* = 0 sample. This spectral feature has been previously associated to the water molecules placed in the second solvation sphere of the hexa-aquo clusters formed by metal ions like the Co^2+^, Ni^2+^, and Zn^2+^ ones in aqueous solution.^48,49^ The absence of this bump in the absorption spectrum of the NiCl_2_·6H_2_O:urea 1:3.5 MDES derives from the presence of only six water molecules per Ni^2+^ ion in the sample and thus too few to complete a second hydration sphere. On the other hand, in the *W* = 26 mixture, there is enough water to form hexa-aquo clusters of Ni^2+^, while the remaining water molecules can populate the second solvation sphere, giving rise to this spectral feature. In addition, in the XANES spectrum of the NiCl_2_·6H_2_O:urea 1:3.5 MDES, a decrease of the white line intensity with respect to the other samples can be observed (Figure 6a), which has been previously associated to the replacement of water molecules by Cl^–^ anions in the Ni^2+^ coordination sphere.^56^ Further insights can be obtained from the comparison between the EXAFS part of the absorption spectra (Figure 6b), which is known to possess a picometric sensitivity on the first neighbor distances.^39−41,49,50,65^ The EXAFS spectra of the three samples are quite similar in the low-*k* region (*k* < 6 Å^–1^), while for higher *k* values, a mismatch in the phase of the oscillation is observed. In particular, the χ(*k*) signal of the NiCl_2_·6H_2_O:urea 1:3.5 MDES shows a shift at lower values of the photoelectron wave vector with respect to the *W* = 26 mixture and to the Ni^2+^ aqueous solution. This differentiation indicates the presence of an additional first neighbor and is fully compatible with the coordination of the chloride anion in the pure MDES, since it has been previously found that this anion coordinates at longer distances with respect to the water molecule.^55,56^

#### EXAFS Results

**Figure 7:**
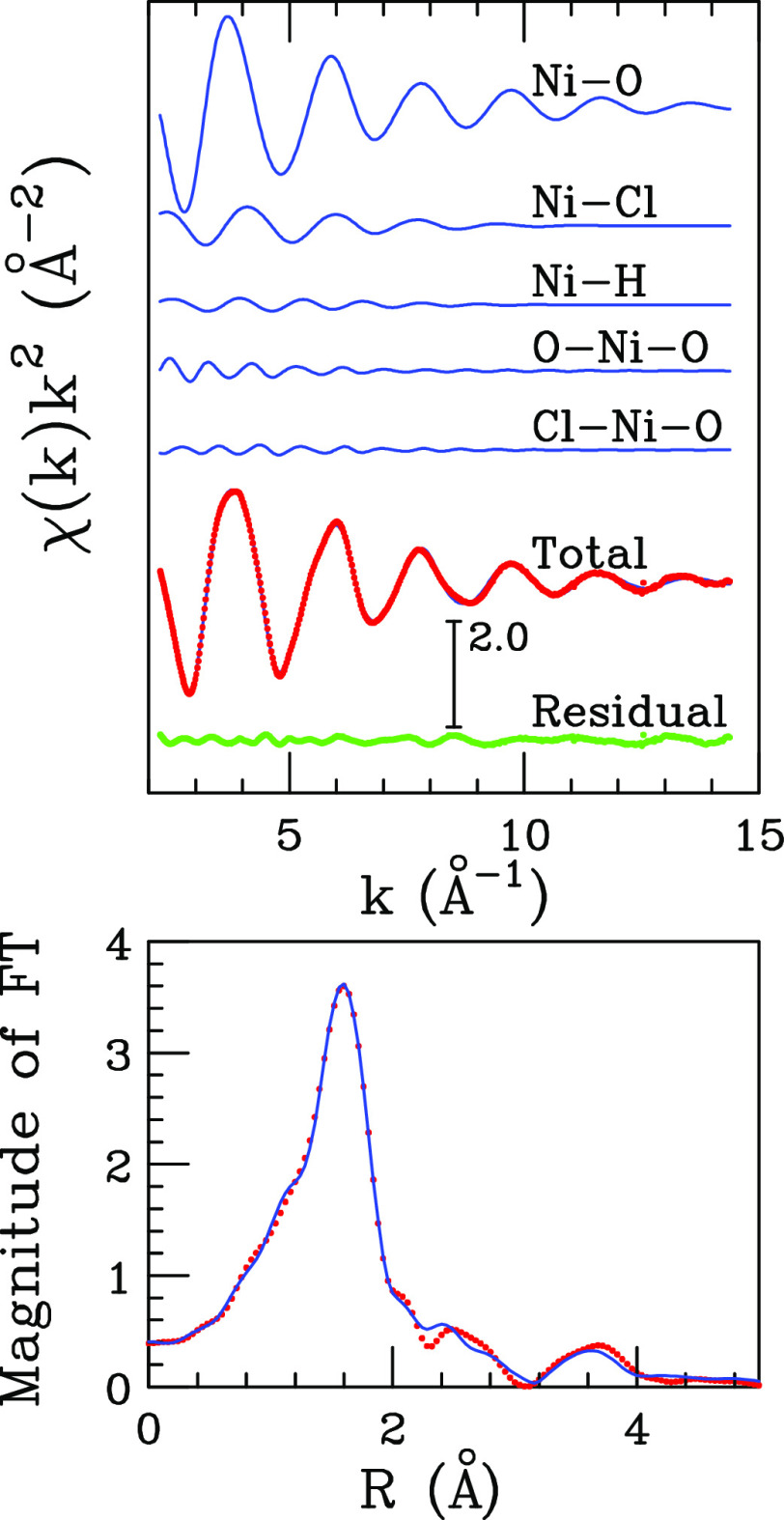
Upper panel: analysis of the Ni K-edge EXAFS spectrum collected on the NiCl_2_·6H_2_O:urea 1:3.5 MDES. From the top to the bottom: Ni–O, Ni–Cl, and Ni-H SS theoretical signals, O–Ni–O and Cl–Ni–O MS theoretical signals, total theoretical spectrum (blue line) compared with the experimental one (red dots), and the resulting residuals (green dots). Lower panel: non-phase shift corrected FT’s of the best-fit EXAFS theoretical signal (blue line) and of the experimental data (red dots). The FT’s have been calculated in the 2.2–13.5 Å^–1^*k*-range.

To have a quantitative determination of the local structure around the Ni^2+^ ion, a fitting procedure of the EXAFS part of the absorption spectrum collected on the NiCl_2_·6H_2_O:urea 1:3.5 MDES has been carried out, and the best-fit results are shown in the upper panel of Figure 7. Here, the two-body theoretical signals Ni–O and Ni–H relative to the water molecule, the Ni–Cl one relative to the chloride anion, and the three-body signals connected with the O–Ni–O and Cl–Ni–O distributions are reported together with the total theoretical contribution compared with the experimental data and the resulting residual. As can be observed, an excellent agreement is obtained between the theoretical and experimental spectra, and the same is true for the corresponding Fourier transformed (FT) spectra shown in the lower panel of Figure 7. The complete list of the optimized structural parameters is reported in Table 1, while the *E*_0_ value resulted to be 0.1 eV below the first inflection point of the experimental spectrum. According to the obtained parameters, the Ni^2+^ ion is coordinated by an average number of 4.8 water molecules, while the coordination number for the chloride anion is 1.2. This result is fully consistent with the average coordination obtained from the MD simulations (Figure 2d) and with the *ab initio* simulations of the UV–vis transitions (Figure 5b). The optimized structural parameters for the Ni–O and Ni–H distributions are in line with those previously determined for Ni^2+^ aqueous solutions.^49−51^ In particular, a Ni–O average distance of 2.06 Å is obtained, while the Ni–Cl distance has been found equal to 2.27 Å (Table 1). On the other hand, the EXAFS fit for the NiCl_2_·6H_2_O:urea:water 1:3.5:26 mixture showed a very good match for a hexa-aquo Ni^2+^ coordination (Figure S5 and Table S6). This result is consistent with the dephase in the EXAFS oscillation observed between the NiCl_2_·6H_2_O:urea 1:3.5 MDES and the *W* = 26 mixture caused by the coordination of the Cl^–^ anion in the pure MDES (Figure 6b). Note that, although the high energy part of the absorption spectrum allows an accurate determination of the first neighbors distances, the coordination numbers are affected by a higher uncertainty. This happens because the EXAFS region is highly affected by the atomic thermal and structural disorder, resulting in a high correlation between the Debye–Waller factor and the coordination number, as both parameters affect the amplitude of the χ(*k*) oscillation.^65,67,68^

**Table 1:** Best-Fit Structural Parameters for the Ni–O, Ni–Cl, and Ni–H SS Paths Obtained from the Analysis of the Ni K-Edge EXAFS Spectrum Collected on the NiCl_2_·6H_2_O:Urea 1:3.5 MDES (*N* Is the Coordination Number, *R* is the Average Distance, σ^2^ Is the Debye–Waller Factor, and β is the Asymmetry Index)

	*N*	*R* (Å)	σ^2^ (Å^2^)	β
Ni–O	4.8(3)	2.06(2)	0.005(2)	0.0(1)
Ni–Cl	1.2(4)	2.27(3)	0.013(3)	0.1(2)
Ni–H	9.6(6)	2.80(4)	0.013(5)	0.0(3)

#### XANES Results

**Figure 8:**
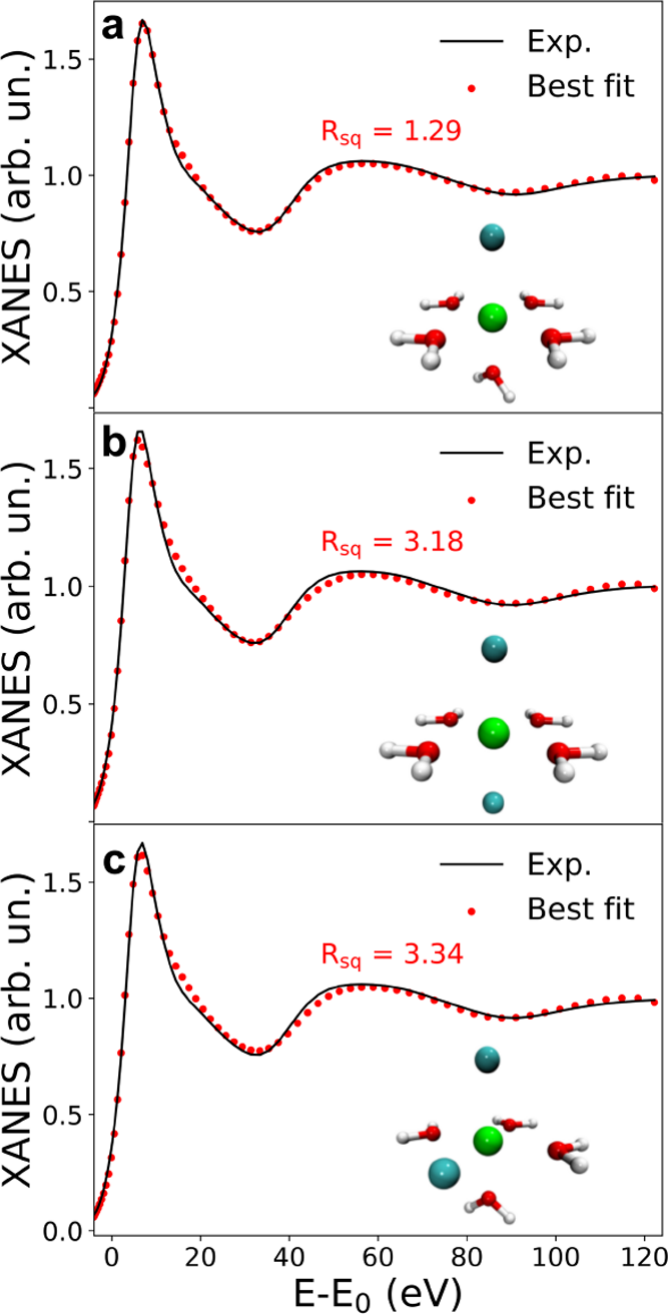
Comparison of the Ni K-edge XANES experimental spectrum collected on the NiCl_2_·6H_2_O:urea 1:3.5 MDES (black line) with the theoretical ones (red dots) optimized for clusters with different Ni^2+^ coordinations: (a) five water molecules and one Cl^–^ anion and four water molecules and two Cl^–^ anions in (b) *trans* and (c) *cis* positions. For each fitting procedure, the obtained residual function *R_sq_* and the optimized cluster are shown as insets.

At variance with the high energy part of the absorption spectrum, the XANES region is less affected by the thermal and structural disorder, allowing a better determination of the first neighbor geometry. To have a more precise determination of the number of chloride anions coordinating the Ni^2+^ ion in the NiCl_2_·6H_2_O:urea 1:3.5 MDES, a fitting procedure of the XANES spectrum has been carried out starting from different octahedral clusters. Given that the MD simulations, the UV–vis data, and the EXAFS analysis suggest that the Ni^2+^ ion is coordinated on average by about one chloride anion, the starting configurations were the [NiCl(H_2_O)_5_]^+^, [NiCl_2_(H_2_O)_4_] (*cis*), and [NiCl_2_(H_2_O)_4_] (*trans*) complexes. The obtained results are shown in Figure 8. As can be observed, the best match between the experimental and the theoretical data is provided by the [NiCl(H_2_O)_5_]^+^ model, while the fits with two chlorides, either in *trans* or in *cis* configuration, result in a worse agreement, as also evidenced by the higher values of the residual function *R_sq_*. In particular, a more pronounced mismatch in the first maximum after the threshold can be observed for the latter coordination modes. The best fit parameters are listed in Table 2, and an average distance between the Ni^2+^ ion and the water oxygen atom of water of 2.05 Å is obtained, while the optimized Ni–Cl distance is 2.30 Å. These values are in good agreement with those obtained from the EXAFS analysis (Table 1), providing the systematic error of XANES analysis on bond lengths.^65^ The analysis of the XANES spectrum of the NiCl_2_·6H_2_O:urea:water 1:3.5:26 mixture has been also carried out, and a good agreement between the theoretical and experimental data has been obtained for the [Ni(H_2_O)_6_]^2+^ cluster (Figure S6 and Table S7). The complementary information provided by the combined analysis of the XANES and EXAFS regions allowed us to find a robust model for the local structure around the Ni^2+^ ion in the studied systems.

**Table 2:** Best-Fit Structural and Non-structural Parameters Obtained from the XANES Analysis of the Ni K-Edge Experimental Spectrum Collected on the NiCl_2_·6H_2_O:Urea 1:3.5 MDES Reported in Figure 8a^a^

*R*_*Ni* – *O*_ (Å)	*R*_*Ni* – *Cl*_ (Å)	*E*_0_ (eV)	*E_F_* (eV)	*E_S_* (eV)	*A_S_*	Γ_*exp*_ (eV)
2.05(2)	2.30(4)	–3.6	–6.1	10.4	9.4	1.4

a *R*_Ni – O_ and *R*_Ni – Cl_ are the Ni–O and Ni–Cl distances, respectively, *E*_0_ is the threshold energy, *E_F_* the Fermi energy, *E_S_* and *A_S_* the plasmon energy onset and amplitude, and Γ_*exp*_ is the experimental resolution.

## Conclusions

In this work, the NiCl_2_·6H_2_O:urea 1:3.5 MDES has been prepared for the first time and it has been found that the eutectic is not obtained with the anhydrous form of the metal salt. The role of water in the MDES formation has been explored by studying NiCl_2_·6H_2_O:urea:water mixtures at different 1:3.5:*W* molar ratios with a multidisciplinary approach, targeting both the local structure around the Ni^2+^ ion and the intermediate-range structural arrangement. MD simulations showed that, in the pure MDES, a close-packing of Ni^2+^ ion clusters forming oligomeric agglomerates is present, which is made possible by the mediation of bridging chloride anions and water molecules. In particular, water coordinating the Ni^2+^ ions can in turn H-bond with further coordinating ligands, acting both as HBD and HBA. This structural arrangement highlighted the fundamental role of water in the MDES formation and explained why the eutectic mixture is obtained only with the hydrated metal salt. On the other hand, the urea molecules poorly coordinate the metal ion and are mostly interspersed in the regions of space among the Ni^2+^ ion clusters, acting as a sort of inner solvent lubricating the oligomeric nanostructure. Such a structural arrangement is disrupted upon the introduction of additional water, which forces the Ni^2+^ ions to move away from each other. This picture is confirmed by the SWAXS data, evidencing a diagnostic prepeak in the WAXS region consistent with the electron density inhomogeneities formed by the Ni–Ni distributions. The area of the water absorption in the NIR region is also coherent with this evolution of the system, showing a tightly bound water population in the NiCl_2_·6H_2_O:urea 1:3.5 MDES while increasing the *W* value turns into a spectral profile resembling more and more that of bulk water. The local structure around the metal ion has been determined by the UV–vis and XAS investigations, which unambiguously showed that the Ni^2+^ ion is coordinated by an average number of one chloride anion and five water molecules in the pure MDES, as evidenced from the *ab initio* simulation of the electronic transitions and from the EXAFS and XANES data analysis. When additional water is added to the MDES, the metal ion solvation sphere is easily saturated to produce a hexa-aquo Ni^2+^ coordination. This multidisciplinary point of view allowed us to probe the MDES structure on different scale lengths and to enlighten the role of water in the eutectic formation, making the information here reported of potential interest for the technological employment of these solvents while also deepening the knowledge about their nature and possibly tackling their fundamental definition.

## Supporting Information Available

Methodological details of the MD simulations, SWAXS data reduction, *ab initio* calculations, EXAFS, and XANES data analysis; additional results for the MD simulations, for the *ab initio* calculations, and for the NIR measurements; and additional EXAFS and XANES data fits (PDF)
