# Supplementary material for: On the Role of Water in the Formation of a Deep Eutectic Solvent Based on NiCl2·6H2O and Urea
Source: Inorg Chem. 2022 May 26;61(23):8843–53. doi: 10.1021/acs.inorgchem.2c00864 (PMC9199011; doi:10.1021/acs.inorgchem.2c00864)
Supplement: Supplementary file 1 — ic2c00864_si_001.pdf [file ic2c00864_si_001.pdf]

Supporting Information

On the Role of Water in the Formation of a Deep Eutectic Solvent based on $NiCl_2$•$6H_2O$ and Urea

Matteo Busato,* Alessandro Tofoni, Giorgia Mannucci, Francesco Tavani, Alessandra Del Giudice, Andrea Colella, Mauro Giustini, and Paola D'Angelo*[a]

[a]Department of Chemistry, University of Rome "La Sapienza", P.le A. Moro 5, 00185, Rome, Italy

*Corresponding author
E-mail:
matteo.busato@uniroma1.it
p.dangelo@uniroma1.it

Pages: S1 – S20
Tables: S1 – S7
Figures: S1 – S6

1. MD simulations

After the energy minimization, each of the systems listed in Table S1 was equilibrated in the NVT ensemble following a heating ramp from 300 to 700 K, staying at high temperature for 10 ns, and gradually cooling down to 300 K, for a total equilibration time of 20 ns. High-temperature equilibrations were previously observed to be recommendable for systems with slow dynamics such as DESs and ionic liquids.[1–3] Production runs were carried out in NVT conditions at 300 K for 50 ns. The temperature was controlled with the Nosé-Hoover thermostat with a relaxation constant of 0.5 ps. The equations of motion were integrated with the leap-frog algorithm with a time step of 1 fs and positions were saved every 1000 steps.

All the simulations were carried out with the Gromacs 2020.6 package,[4] while the VMD 1.9.3 software was used for trajectories visualization.[5] In-house codes were employed for the calculation of the combined distribution function (CDF) and for the instantaneous coordination numbers.

2. SWAXS data reduction

The two-dimensional scattering patterns obtained for the $NiCl_2$•$6H_2O$:urea:water mixtures at different 1:3.5:*W* molar ratios were subtracted for the dark counts, and then masked, azimuthally averaged, and normalized for transmitted beam intensity, exposure time, and subtended solid angle per pixel, by using the FoxTrot software developed at SOLEIL. The one-dimensional intensity *vs*. *q* profiles were then subtracted for the contribution of the empty Kapton windows and put in absolute scale units (cm^{-1}) by dividing for the approximate sample thickness calculated by means of the experimental transmission and the linear absorption coefficient estimated on the basis of the chemical formula using the Advanced Photon Source web utility.[6] The different angular ranges were merged using the SAXSutilities tool.[7]

3. *Ab initio* calculations

The electronic transitions were simulated from *ab initio* calculations for octahedral clusters with different Ni^{2+} coordination, namely $[Ni(H_2O)_6]^{2+}$, $[NiCl(H_2O)_5]^{+}$, $[NiCl_2(H_2O)_4]$ (*cis*), $[NiCl_2(H_2O)_4]$ (*trans*), $[NiCl_3(H_2O)_3]^{-}$, and $[NiCl_4(H_2O)_2]^{2-}$. Only the *mer* isomer and the isomer with the two water molecules in *trans* position were considered for $[NiCl_3(H_2O)_3]^{-}$ and $[NiCl_4(H_2O)_2]^{2-}$, respectively. Geometry optimizations were carried out at the unrestricted density functional theory (DFT) level in gas phase with the B3LYP functional[8,9] and the aug-cc-PVDZ basis set for all atoms,[10–14] using the ORCA 4.2 code.[15] For the sake of comparison, optimizations of the same species were performed also with the introduction of implicit solvent effects with the PCM method[16] selecting water as a medium. In this case, the 6-31+G(*d*,*p*) basis set was employed and the calculations were carried out with the Gaussian 09 code[17] due to lack of convergence issues with the former level of theory after the introduction of the solvent effects. Note that water was selected as implicit solvent since the parameters for the studied MDES are not available. In all cases, a vibrational analysis was carried out to verify the absence of imaginary frequencies and that the stationary points were true minima.

The absorption spectra were obtained with gas-phase calculations on the geometries optimized both in gas-phase and in PCM water at the complete active space self-consistent field (CASSCF) level of theory supported by the strongly correlated N-electron valence state perturbation theory (SC-NEVPT2)[18] using the ORCA 4.2 code.[15] In the SC-NEVPT2 method, a CASSCF(n_{el}, n_{orb}) wavefunction is first computed as a Full-CI expansion in the active space defined by the n_{el} valence electrons in the n_{orb} active orbitals, and the CI problem is then solved for an arbitrary number of roots. Each root is then considered as a zero-order wave function $\Psi_m^{(0)}$ perturbed by the wavefunctions $\Psi_l^{(k)}$, where k is the number of electrons promoted to or removed from the active space and l specifies a fixed occupation pattern of the inactive orbitals.[19] The absorption spectrum can then be computed *via* simple dipole integrals between the ground and the excited states. This method was previously demonstrated to provide accurate results for the calculation of the absorption spectra of open-shell systems and in particular for Ni^{2+} complexes.[20] In this work, all of the possible 9 excited triplet states were included in the calculations, while singlet states corresponding to dipole forbidden transitions were not considered. Ten roots were calculated in both the (8,5) and (14,8) active spaces. The triple-ζ polarized ma-def2-PVTZ basis set with diffuse

functions was used for all atoms.[21] No significant differences in the obtained transitions were observed including the PCM solvent instead of the gas phase conditions.

4. EXAFS data analysis

The analysis of the EXAFS part of the absorption spectra collected on the $NiCl_2 \bullet 6H_2O$:urea:water 1:3.5:*W* mixtures with *W* = 0 and 26 was carried out with the GNXAS program.[22,23] The amplitude function $A(k, r)$ and phase shifts $\varphi(k, r)$ have been calculated from clusters with fixed geometry within the muffin-tin (MT) approximation and the Hedin-Lundqvist (HL) scheme for the exchange-correlation self-energy of the photoelectron in the final state, which allow one to take into account the inelastic losses intrinsically.[24] Theoretical signals associated with *n*-body distribution functions have been calculated in accordance with the multiple-scattering (MS) theory and summed in order to reconstruct the total theoretical contribution. Each two-body distribution has been modeled as a Γ-like function depending on four structural parameters, namely the coordination number *N*, the average distance *R*, the Debye-Waller factor σ^2, and the asymmetry index *β*, which have been optimized during the fitting procedure to obtain the best agreement with the experimental data.

The EXAFS spectrum of the $NiCl_2 \bullet 6H_2O$:urea 1:3.5 MDES was analyzed with a double-step strategy. Two-body single scattering (SS) signals have been calculated to take into account the first shell chlorine ions (Ni-Cl) and water molecules. For the latter ones, both the contribution related to the oxygen (Ni-O) and hydrogen atoms (Ni-H) have been included. In the first step, the corresponding N values of the chlorine anion and of water have been optimized by keeping the total amount of first neighbors fixed to 6, being known from the UV-Vis data that the Ni^{2+} ion was hexacoordinated in this system (see the main text). In the second step, the coordination numbers were constrained to the optimized values and the three-body MS terms connected with the O-Ni-O and Cl-Ni-O collinear paths were also included. The EXAFS spectrum of the $NiCl_2 \bullet 6H_2O$:urea:water 1:3.5:26 mixture was fitted by taking into account a hexa-aquo coordination of the Ni^{2+} ion. Ni-O and Ni-H signals connected with six water molecules were calculated, and the MS contribution related to the collinear O-Ni-O paths was also included.

For all the fitting procedures, least-squares minimizations have been carried out in the 2.2 - 14.4 Å^{-1} k-range on the raw data directly, without preliminary background subtraction and Fourier filtering, by optimizing all the structural parameters. Non-structural parameters have been also optimized, namely the Ni K-edge ionization energy E_0, and the energy positions and amplitudes of the double-electron excitation channels KM_1 and $KM_{2,3}$. The inclusion of the double-electron excitations allowed us to keep the S_0^2 amplitude reduction factor constrained between 0.95 and 1.00. The MT radii for the Ni, O and H atoms were respectively 1.6, 0.9, and 0.2 Å, as previously employed.[25,26] A radius of 1.2 Å for the Cl atom was chosen to obtain a 20 % overlap with the nickel adjacent MT sphere.

5. XANES data analysis

The analysis of the XANES part of the absorption spectra collected on the $NiCl_2$•$6H_2O$:urea:water 1:3.5:W mixtures with W = 0 and 26 was carried out with the MXAN code.[27] The potential has been calculated in the framework of the MT approximation using a complex optical potential, based on the local density approximation of the self-energy of the excited photoelectron.[28] The real part of the self-energy was calculated with the HL scheme,[24] since the full complex HL potential is known to introduce a relevant over-damping at low energies. Therefore, the inelastic losses were accounted for with a phenomenological approach by convolution of the theoretical spectrum with a Lorentzian function having an energy-dependent width of the form $\Gamma_{tot}(E) = \Gamma_c + \Gamma_{mfp}(E)$. Γ_c is the core-hole lifetime, while the energy-dependent term $\Gamma_{mfp}(E)$ represents all the intrinsic and extrinsic inelastic processes. $\Gamma_{mfp}(E)$ is zero below an onset energy E_s, that in extended systems corresponds to the plasmon excitation energy, and begins to increase from a value A_s following the universal form of the mean-free path in solids.[29] The numerical values of E_s and A_s are derived at each step of computation (*i.e.*, for each geometrical configuration) using a Monte Carlo fit. The experimental resolution is taken into account by a further convolution with an energy independent Gaussian function.

Least-squares fits have been carried out by optimizing the geometry of the starting models with the minimization of a residual function R_{sq} defined as:

$$R_{sq} = \frac{\sum_{i=1}^{m} w_i (y_i^{th} - y_i^{exp})^2}{\varepsilon_i^2 \sum_{i=1}^{m} w_i}$$

where m is the number of data points, y_i^{th} and y_i^{exp} are the theoretical and experimental values of the absorption, respectively, ε_i is the individual error in the experimental data set, and w_i is a statistical weight. For the XANES data fit of the $NiCl_2$•$6H_2O$:urea 1:3.5 MDES, three octahedral clusters with different Ni^{2+} coordination spheres were employed as starting models, *i.e.*, $[NiCl(H_2O)_5]^+$, $[NiCl_2(H_2O)_4]$ (*cis*), and $[NiCl_2(H_2O)_4]$ (*trans*). The spectrum of the $NiCl_2$•$6H_2O$:urea:water 1:3.5:26 mixture was analyzed starting from the $[Ni(H_2O)_6]^{2+}$ complex. In addition, five non-structural parameters were optimized, namely the experimental resolution Γ_{exp}, the Fermi energy level E_F, the threshold energy E_0, the energy and amplitude of the plasmon E_s and A_s.

Table S1. Number of species, box dimensions, and densities of the simulated MD systems for the $NiCl_2$•$6H_2O$:urea:water mixtures at different 1:3.5:*W* molar ratios.

W	Ni^{2+}	Cl^-	Urea	H_2O	Box edge (Å)	d (g cm^{-3})†
**0**	253	506	886	1518	49.99	1.506
**4**	204	408	714	2040	50.04	(1.406)
**8**	170	340	595	2380	49.96	(1.340)
**12**	146	292	511	2628	49.96	(1.291)
**16**	128	256	448	2816	49.96	(1.255)
**26**	98	196	343	3136	49.98	(1.194)
**50**	63	126	221	3528	50.08	(1.123)

†Experimental density of the $NiCl_2$•$6H_2O$:urea 1:3.5 MDES obtained by weighting 1 mL of sample in a volumetric flask. The values in parentheses are the ideal densities for the $NiCl_2$•$6H_2O$:urea:water 1:3.5:*W* mixtures calculated with Eq. 1 of the manuscript.

Table S2. Molality of the Ni^{2+} ion in the $NiCl_2$•$6H_2O$:urea:water mixtures at different 1:3.5:*W* molar ratios employed for the UV-Vis measurements.

W	$m_{Ni^{2+}}$ (mol kg^{-1})
**0**	2.57
**4**	2.16
**8**	1.87
**12**	1.65
**16**	1.48
**26**	1.17
**50**	0.78

Table S3. Maxima positions for the $^3A_{2g}$(F) → $^3T_{1g}$(P), $^3A_{2g}$(F) →$^3T_{1g}$(F), and $^3A_{2g}$(F) →^1E_g(D) transitions in the experimental UV-Vis absorption spectra of the $NiCl_2$•$6H_2O$:urea:water mixtures at different 1:3.5:*W* molar ratios and of the 50 mM $NiCl_2$ aqueous solution.

W	λ_{max} (nm)		
	$^3A_{2g}$(F) →$^3T_{1g}$(P)	$^3A_{2g}$(F) →$^3T_{1g}$(F)	$^3A_{2g}$(F) →^1E_g(D)
**0**	414	690	756
**4**	407	674	740
**8**	404	668	738
**12**	401	662	734
**16**	400	662	731
**26**	398	659	728
**50**	397	654	723
**Ni^{2+} in H_2O**	394	654	718

Table S4. Representative bond distances (Å) and bond angles (degrees) for the Ni^{2+} ion clusters optimized at the uB3LYP/aug-cc-PVDZ level in gas phase and at the uB3LYP/6-31+G(*d*,*p*) level in PCM water.

	**$[Ni(H2O)_6]^{2+}$**	
	**PCM(Water)/6-31+G(*d,p*)**	**Gas phase/aug-cc-PVDZ**
r(Ni-O1)	2.072	2.083
r(Ni-O2)	2.077	2.084
r(Ni-O3)	2.073	2.084
r(Ni-O4)	2.068	2.078
r(Ni-O5)	2.076	2.083
r(Ni-O6)	2.067	2.078
∠(O-Ni-O) (1)	173.7	180.0
∠(O-Ni-O) (2)	179.6	179.9
∠(O-Ni-O) (3)	173.9	178.9
	**$[Ni(H2O)_5Cl]^{+}$**	
	**PCM(Water)/6-31+G(*d,p*)**	**Gas phase/aug-cc-PVDZ**
r(Ni-O1)	2.100	2.122
r(Ni-O2)	2.095	2.116
r(Ni-O3)	2.107	2.109
r(Ni-O4)	2.086	2.106
r(Ni-O5)	2.098	2.124
r(Ni-Cl)	2.349	2.289
∠(O-Ni-O) (1)	178.3	176.5
∠(O-Ni-O) (2)	170.5	177.7
∠(O-Ni-Cl)	177.6	178.9
	**$[Ni(H2O)_4Cl_2]$ (*cis*)**	
	**PCM(Water)/6-31+G(*d,p*)**	**Gas phase/aug-cc-PVDZ**
r(Ni-O1)	2.109	2.151
r(Ni-O2)	2.131	2.166
r(Ni-O3)	2.130	2.128
r(Ni-O4)	2.109	2.130
r(Ni-Cl1)	2.393	2.365
r(Ni-Cl2)	2.391	2.362
∠(O-Ni-O)	176.7	173.3
∠(O-Ni-Cl) (1)	170.9	170.8
∠(O-Ni-Cl) (2)	171.2	170.7
	**$[Ni(H2O)_4Cl_2]$ (*trans*)**	
	**PCM(Water)/6-31+G(*d,p*)**	**Gas phase/aug-cc-PVDZ**
r(Ni-O1)	2.105	2.156
r(Ni-O2)	2.114	2.136
r(Ni-O3)	2.109	2.111
r(Ni-O4)	2.114	2.153
r(Ni-Cl1)	2.392	2.344
r(Ni-Cl2)	2.388	2.367
∠(O-Ni-O) (1)	177.3	171.4
∠(O-Ni-O) (2)	178.0	170.3
∠(Cl-Ni-Cl)	178.3	170.2
	**$[Ni(H2O)_3Cl_3]^{-}$**	
	**PCM(Water)/6-31+G(*d,p*)**	**Gas phase/aug-cc-PVDZ**
r(Ni-O1)	2.125	2.214
r(Ni-O2)	2.133	2.207
r(Ni-O3)	2.121	2.158
r(Ni-Cl1)	2.449	2.388
r(Ni-Cl2)	2.412	2.456
r(Ni-Cl3)	2.463	2.401
∠(O-Ni-O)	179.8	162.9
∠(O-Ni-Cl)	178.5	176.9
∠(Cl-Ni-Cl)	167.2	163.9
	**$[Ni(H2O)_2Cl_4]^{2-}$**	
	**PCM(Water)/ 6-31+G(*d,p*)**	**Gas phase/aug-cc-PVDZ**

r(Ni-O1)	2.129	2.197
r(Ni-O2)	2.131	2.196
r(Ni-Cl1)	2.492	2.469
r(Ni-Cl2)	2.546	2.562
r(Ni-Cl3)	2.498	2.559
r(Ni-Cl4)	2.441	2.497
∠(O-Ni-O)	170.5	179.9
∠(Cl-Ni-Cl) (1)	178.4	180.0
∠(Cl-Ni-Cl) (2)	178.6	179.9

Table S5. CASSCF states, vertical transition energy ΔE (eV, nm in parenthesis) and oscillator strength *f* of the two most intense transitions in the 350 - 450 nm and 600 - 1050 nm regions obtained from the gas phase CASSCF/NEVPT2 calculations with the (8,5) and (14,8) active spaces for the Ni^{2+} clusters optimized at the uB3LYP/aug-cc-PVDZ level in gas phase and at the uB3LYP/6-31+G(*d,p*) level in PCM water.

**$[Ni(H2O)_6]^{2+}$**		
**PCM(Water)/6-31+G(*d,p*) (8,5)**		
**State**	**ΔE**	***f***
7	3.401 (364)	1.99×10^{-7}
6	1.844 (672)	5.40×10^{-8}
**$[Ni(H2O)_6]^{2+}$**		
**PCM(Water)/6-31+G(*d,p*) (14,8)**		
**State**	**ΔE**	***f***
7	3.340 (371)	1.91×10^{-7}
6	1.818 (682)	5.30×10^{-8}
**$[Ni(H2O)_6]^{2+}$**		
**Gas phase/aug-cc-PVDZ (8,5)**		
**State**	**ΔE**	***f***
9	3.291 (377)	$6..00\times 10^{-9}$
4	1.598 (776)	4.00×10^{-9}
**$[Ni(H2O)_6]^{2+}$**		
**Gas phase/aug-cc-PVDZ (14,8)**		
**State**	**ΔE**	***f***
9	3.269 (379)	1.00×10^{-9}
4	1.588 (781)	4.00×10^{-9}
**$[Ni(H2O)_5Cl]^{+}$**		
**PCM(Water)/6-31+G(*d,p*) (8,5)**		
**State**	**ΔE**	***f***
7	3.293 (377)	7.97×10^{-7}
6	1.740 (713)	1.61×10^{-7}
**$[Ni(H2O)_5Cl]^{+}$**		
**PCM(Water)/6-31+G(*d,p*) (14,8)**		
**State**	**ΔE**	***f***
7	3.190 (720)	2.63×10^{-6}
6	1.722 (389)	6.34×10^{-7}

$[Ni(H2O)_5Cl]^+$		
**Gas phase/aug-cc-PVDZ (8,5)**		
**State**	**ΔE**	***f***
7	3.235 (383)	2.03×10^{-6}
4	1.650 (751)	5.72×10^{-7}

$[Ni(H2O)_5Cl]^+$		
**Gas phase/aug-cc-PVDZ (14,8)**		
**State**	**ΔE**	***f***
7	3.122 (397)	6.09×10^{-6}
4	1.593 (778)	2.18×10^{-7}

$[Ni(H2O)_4Cl_2]$ (*cis*)		
**PCM(Water)/6-31+G(*d,p*) (8,5)**		
**State**	**ΔE**	***f***
7	3.186 (389)	5.47×10^{-6}
5	1.605 (773)	1.77×10^{-6}

$[Ni(H2O)_4Cl_2]$ (*cis*)		
**PCM(Water)/6-31+G(*d,p*) (14,8)**		
**State**	**ΔE**	***f***
7	3.104 (399)	6.06×10^{-6}
5	1.580 (785)	1.52×10^{-6}

$[Ni(H2O)_4Cl_2]$ (*cis*)		
**Gas phase/aug-cc-PVDZ (8,5)**		
**State**	**ΔE**	***f***
8	3.178 (390)	5.19×10^{-6}
5	1.614 (768)	1.44×10^{-6}

$[Ni(H2O)_4Cl_2]$ (*cis*)		
**Gas phase/aug-cc-PVDZ (14,8)**		
**State**	**ΔE**	***f***
8	3.081 (402)	4.99×10^{-6}
5	1.575 (787)	1.27×10^{-6}

$[Ni(H2O)_4Cl_2]$ (*trans*)		
**PCM(Water)/6-31+G(*d,p*) (8,5)**		
**State**	**ΔE**	***f***
9	3.290 (754)	4.95×10^{-7}
6	1.644 (377)	1.16×10^{-7}

$[Ni(H2O)_4Cl_2]$ (*trans*)		
**PCM(Water)/6-31+G(*d,p*) (14,8)**		
**State**	**ΔE**	***f***
9	3.262 (380)	4.63×10^{-7}
6	1.618 (733)	1.09×10^{-7}

$[Ni(H2O)_4Cl_2]$ (*trans*)		
**Gas phase/aug-cc-PVDZ (8,5)**		
**State**	**ΔE**	***f***
8	3.159 (393)	1.23×10^{-6}
4	1.510 (821)	3.78×10^{-7}

$[Ni(H2O)_4Cl_2]$ (*trans*)
**Gas phase/aug-cc-PVDZ (14,8)**

State	ΔE	*f*
8	3.076 (403)	1.29×10^{-6}
4	1.488 (834)	3.47×10^{-7}
	**$[Ni(H2O)_3Cl_3]^-$**	
	**PCM(Water)/6-31+G(*d,p*) (8,5)**	
**State**	**ΔE**	***f***
7	3.072 (404)	3.72×10^{-6}
6	1.498 (828)	1.19×10^{-6}
	**$[Ni(H2O)_3Cl_3]^-$**	
	**PCM(Water)/6-31+G(*d,p*) (14,8)**	
**State**	**ΔE**	***f***
7	3.004 (838)	5.57×10^{-6}
6	1.480 (413)	1.13×10^{-6}
	**$[Ni(H2O)_3Cl_3]^-$**	
	**Gas phase/aug-cc-PVDZ (8,5)**	
**State**	**ΔE**	***f***
7	2.945 (892)	7.76×10^{-6}
6	1.390 (420)	9.85×10^{-7}
	**$[Ni(H2O)_3Cl_3]^-$**	
	**Gas phase/aug-cc-PVDZ (14,8)**	
**State**	**ΔE**	***f***
7	2.945 (921)	6.97×10^{-6}
6	1.390 (437)	8.87×10^{-7}
	**$[Ni(H2O)_2Cl_4]^{2-}$**	
	**PCM(Water)/6-31+G(*d,p*) (8,5)**	
**State**	**ΔE**	***f***
9	2.992 (415)	3.04×10^{-6}
4	1.283 (966)	6.05×10^{-7}
	**$[Ni(H2O)_2Cl_4]^{2-}$**	
	**PCM(Water)/6-31+G(*d,p*) (14,8)**	
**State**	**ΔE**	***f***
9	2.974 (417)	3.28×10^{-6}
4	1.279 (969)	5.89×10^{-7}
	**$[Ni(H2O)_2Cl_4]^{2-}$**	
	**Gas phase/aug-cc-PVDZ (8,5)**	
**State**	**ΔE**	***f***
8	2.916 (425)	1.9×10^{-8}
6	1.275 (977)	2.0×10^{-9}
	**$[Ni(H2O)_2Cl_4]^{2-}$**	
	**Gas phase/aug-cc-PVDZ (14,8)**	
**State**	**ΔE**	***f***
8	2.904 (427)	3.8×10^{-8}
6	1.275 (977)	2.0×10^{-9}

Table S6. Best-fit structural parameters for the Ni-O and Ni-H SS paths obtained from the analysis of the Ni K-edge EXAFS spectrum collected on the $NiCl_2$•$6H_2O$:urea:water mixture at 1:3.5:26 molar ratio.†

	N	*R* (Å)	σ^2 (Å^2)	β
**Ni-O**	6.0(3)	2.06(2)	0.004(2)	0.0(1)
**Ni-H**	12.0(6)	2.82(4)	0.011(4)	0.0(2)

†*N* is the coordination number, *R* the average distance, σ^2 the Debye-Waller factor, and β the asymmetry index.

Table S7. Best-fit structural and non-structural parameters obtained from the XANES analysis of the Ni K-edge experimental spectrum collected on the $NiCl_2$•$6H_2O$:urea:water mixture at 1:3.5:26 molar ratio.†

R_{Ni-O} (Å)	E_0 (eV)	E_F (eV)	E_S (eV)	A_S	Γ_{exp} (eV)
2.03(3)	-4.1	-5.6	11.8	8.8	1.0

†R_{Ni-O} is the Ni-O distance, E_0 is the threshold energy, E_F the Fermi energy, E_S and A_S the plasmon energy onset and amplitude, and Γ_{exp} the experimental resolution.

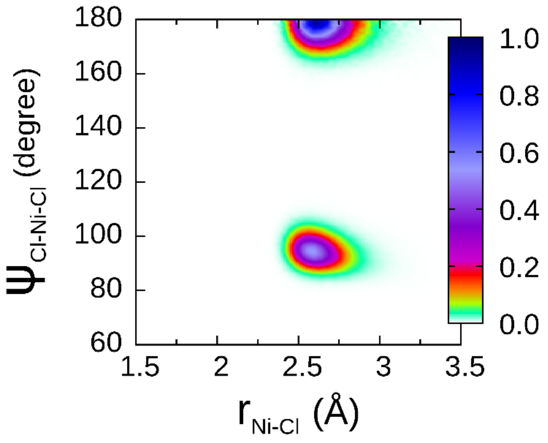

Figure S1. Contour plot of the CDF between the Ni-Cl distances and the Cl-Ni-Cl angles as calculated from the MD simulation for the $NiCl_2$•$6H_2O$:urea 1:3.5 MDES. The box on the right side defines the probability color scale of finding the inspected ion at that distance and angle, normalized to one.

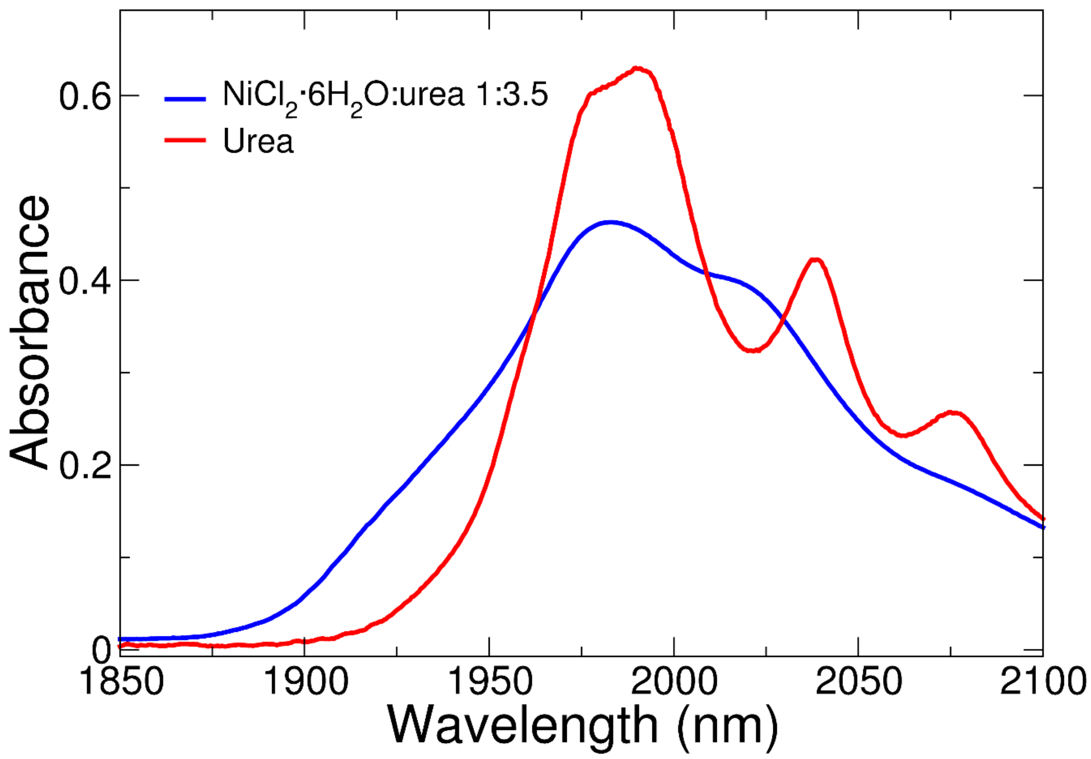

Figure S2. Rescaled NIR absorption spectrum collected on a solid pellet of urea in KBr (red line) compared to the NIR spectrum of the $NiCl_2 \cdot 6H_2O$:urea 1:3.5 MDES (blue line).

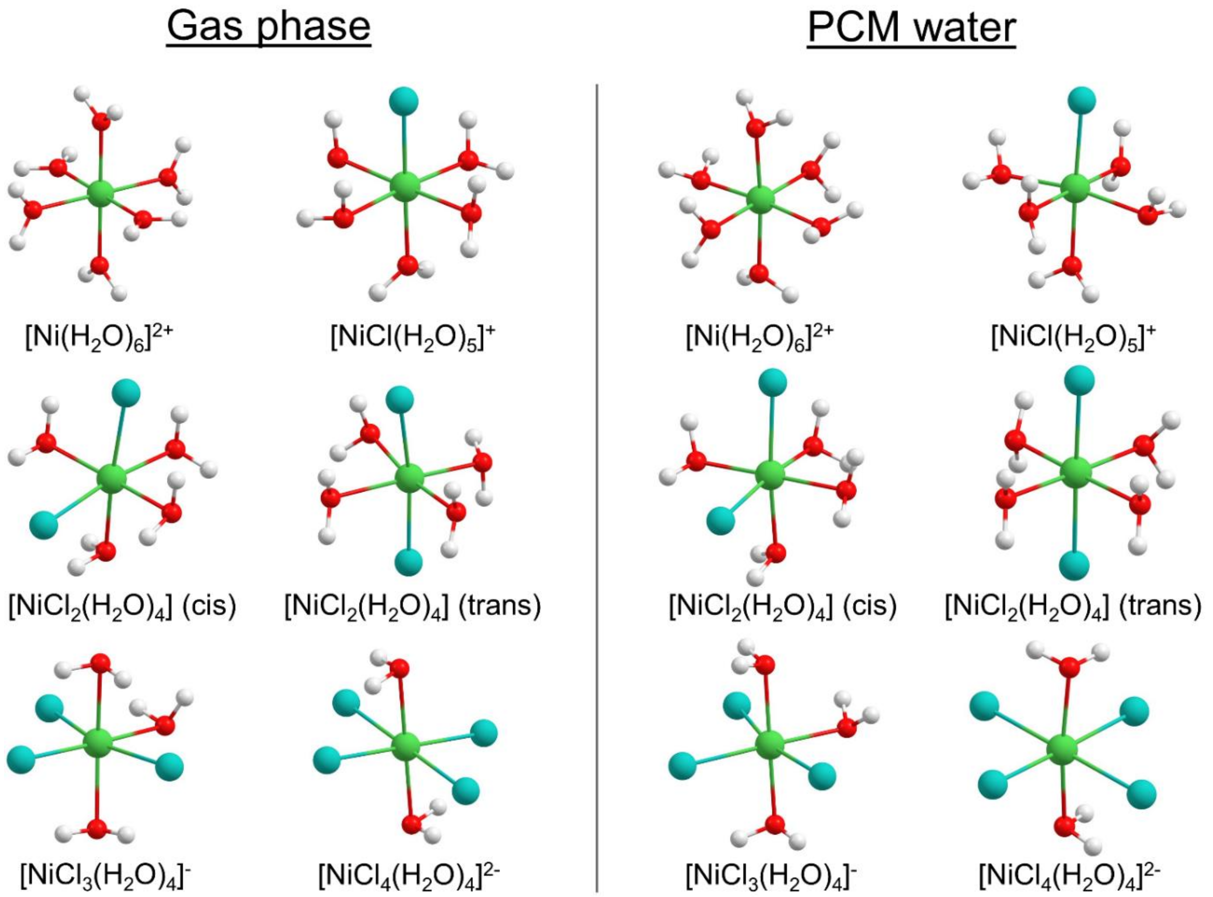

Figure S3. Minimum energy structures of the octahedral Ni^{2+} clusters with different number of chloride anions and water molecules optimized at the uB3LYP/aug-cc-PVDZ level in gas phase (left panel) and at the uB3LYP/6-31+G(*d*,*p*) level in PCM water (right panel).

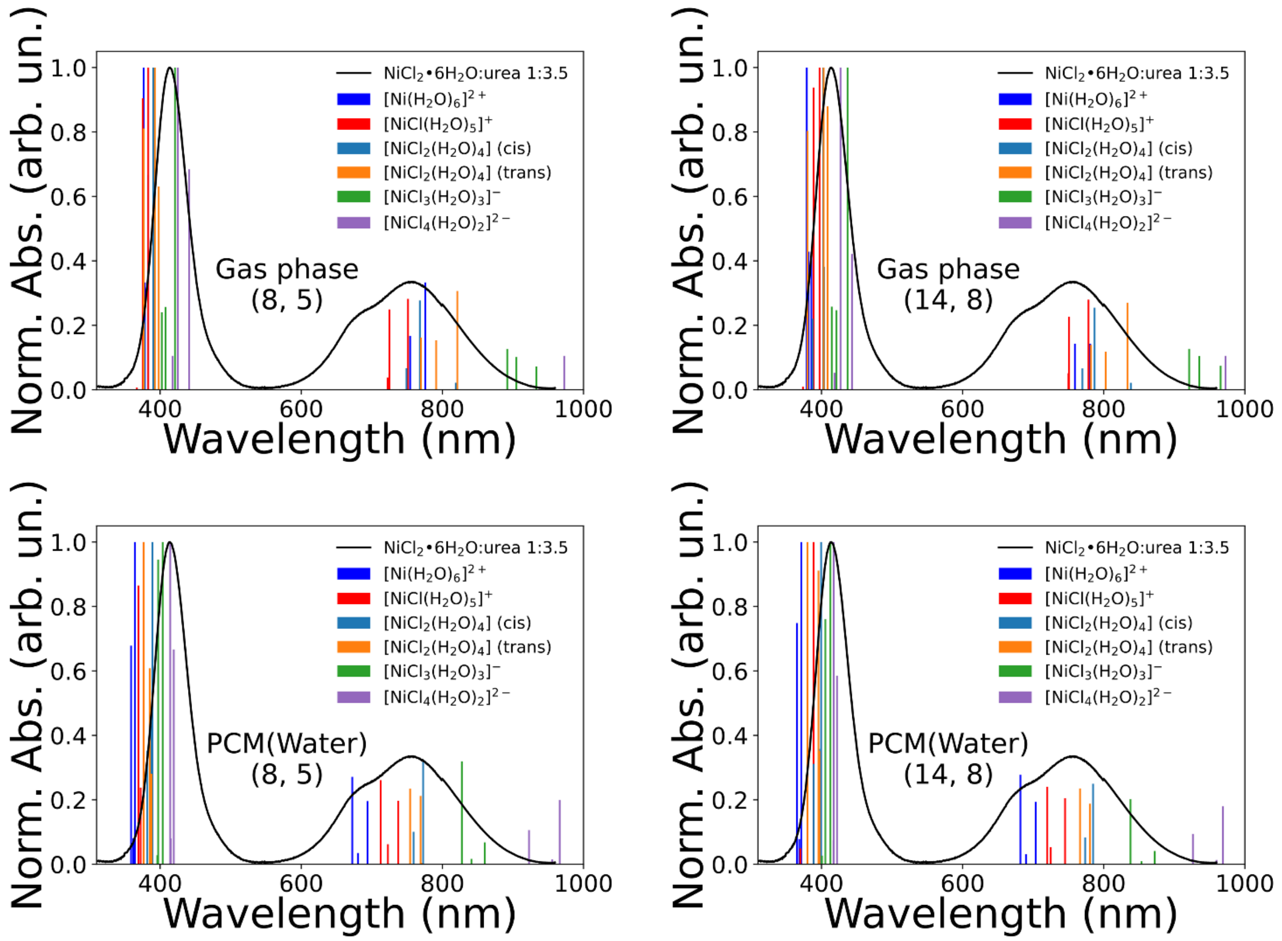

Figure S4. Simulated electronic transitions at the CASSCF/NEVPT2 level of theory from single-point gas phase calculations with (8,5) and (14,8) active spaces (left and right panels, respectively) on the clusters optimized in gas phase (top panels) and PCM water (bottom panels) shown in Figure S3. The experimental UV-Vis absorption spectrum collected on the $NiCl_2$•$6H_2O$:urea 1:3.5 MDES (black line) is reported for comparison.

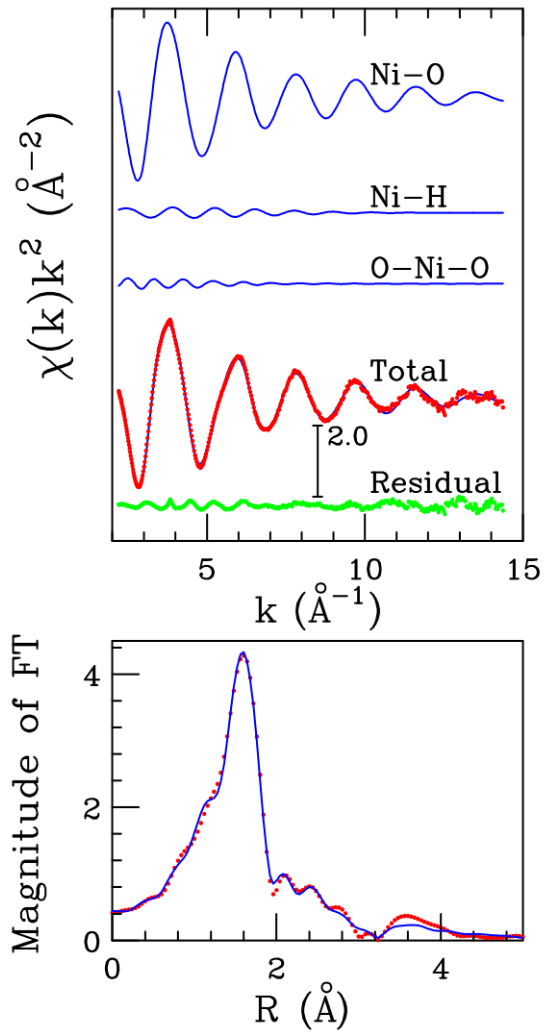

Figure S5. Upper panel: analysis of the Ni K-edge EXAFS spectrum collected on the $NiCl_2$•$6H_2O$:urea:water mixture at 1:3.5:26 molar ratio. From the top to the bottom: Ni-O and Ni-H SS theoretical signals, O-Ni-O MS theoretical signal, total theoretical spectrum (blue line) compared with the experimental one (red dots) and the resulting residuals (green dots). Lower panel: non-phase shift corrected Fourier Transforms (FT's) of the best-fit EXAFS theoretical signal (blue line) and of the experimental data (red dots). The FT's have been calculated in the 2.2 – 13.5 Å^{-1} *k*-range.

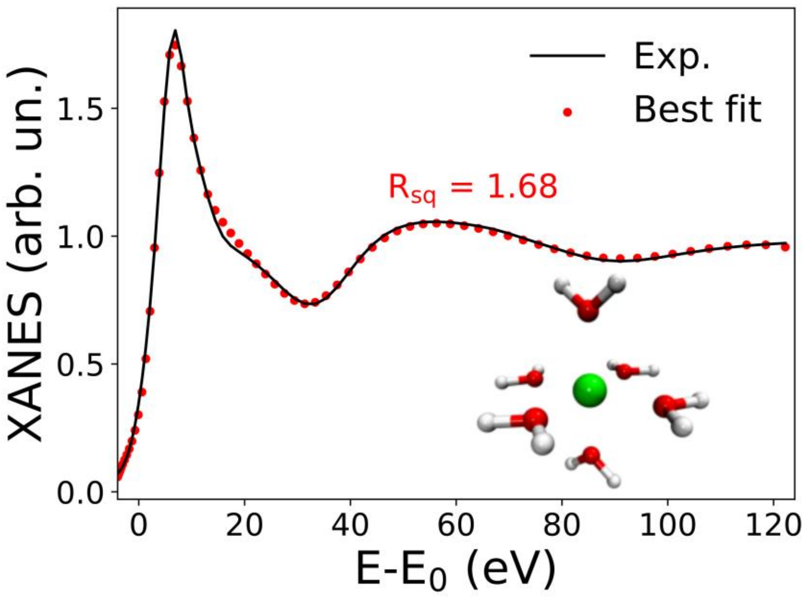

Figure S6. Comparison of the Ni K-edge XANES experimental spectrum collected on the $NiCl_2$•$6H_2O$:urea:water mixture at 1:3.5:26 molar ratio (black line) with the theoretical one (red dots) optimized for the hexa-aquo coordination of the Ni^{2+} ion. The obtained residual function R_{sq} and the optimized cluster are shown as insets.

References

1. M. Busato, P. D’Angelo, A. Lapi, M. Tolazzi and A. Melchior, Solvation of Co^{2+} ion in 1-butyl-3-methylimidazolium bis(trifluoromethylsulfonyl)imide ionic liquid: A molecular dynamics and X-ray absorption study, *J. Mol. Liq.*, 2020, **299**, 112120.
2. M. Busato, P. D’Angelo and A. Melchior, Solvation of Zn^{2+} ion in 1-alkyl-3-methylimidazolium bis(trifluoromethylsulfonyl)imide ionic liquids: a molecular dynamics and X-Ray absorption study, *Phys. Chem. Chem. Phys.*, 2019, **21**, 6958–6969.
3. V. Migliorati, A. Gibiino, A. Lapi, M. Busato and P. D’Angelo, On the Coordination Chemistry of the lanthanum(III) Nitrate Salt in EAN/MeOH Mixtures, *Inorg. Chem.*, 2021, **60**, 10674–10685.
4. M. J. Abraham, T. Murtola, R. Schulz, S. Páll, J. C. Smith, B. Hess and E. Lindahl, GROMACS: High performance molecular simulations through multi-level parallelism from laptops to supercomputers, *SoftwareX*, 2015, **1**–**2**, 19–25.
5. W. Humphrey, A. Dalke and K. Schulten, VMD: Visual molecular dynamics, *J. Mol.*

Graph., 1996, **14**, 33–38.
6 Advanced Photon Source, https://11bm.xray.aps.anl.gov/absorb/absorb.php, (accessed 1 July 2021).
7 M. Sztucki and T. Narayanan, in *Journal of Applied Crystallography*, 2007, vol. 40, pp. s459–s462.
8 A. D. Becke, A new mixing of Hartree–Fock and local density-functional theories, *J. Chem. Phys.*, 1993, **98**, 1372–1377.
9 C. Lee, W. Yang and R. G. Parr, Development of the Colle-Salvetti correlation-energy formula into a functional of the electron density, *Phys. Rev. B*, 1988, **37**, 785–789.
10 T. H. Dunning, Gaussian basis sets for use in correlated molecular calculations. I. The atoms boron through neon and hydrogen, *J. Chem. Phys.*, 1989, **90**, 1007–1023.
11 R. A. Kendall, T. H. Dunning and R. J. Harrison, Electron affinities of the first-row atoms revisited. Systematic basis sets and wave functions, *J. Chem. Phys.*, 1992, **96**, 6796–6806.
12 D. E. Woon and T. H. Dunning, Gaussian basis sets for use in correlated molecular calculations. III. The atoms aluminum through argon, *J. Chem. Phys.*, 1993, **98**, 1358–1371.
13 N. B. Balabanov and K. A. Peterson, Systematically convergent basis sets for transition metals. I. All-electron correlation consistent basis sets for the 3d elements Sc–Zn, *J. Chem. Phys.*, 2005, **123**, 64107.
14 N. B. Balabanov and K. A. Peterson, Basis set limit electronic excitation energies, ionization potentials, and electron affinities for the 3d transition metal atoms: Coupled cluster and multireference methods, *J. Chem. Phys.*, 2006, **125**, 74110.
15 F. Neese, F. Wennmohs, U. Becker and C. Riplinger, The ORCA quantum chemistry program package, *J. Chem. Phys.*, 2020, **152**, 224108.
16 J. Tomasi, B. Mennucci and R. Cammi, Quantum Mechanical Continuum Solvation Models, *Chem. Rev.*, 2005, **105**, 2999–3094.
17 M. J. Frisch, G. W. Trucks, H. B. Schlegel, G. E. Scuseria, M. A. Robb, J. R. Cheeseman, G. Scalmani, V. Barone, G. A. Petersson, H. Nakatsuji, X. Li, M. Caricato, A. V Marenich, J. Bloino, B. G. Janesko, R. Gomperts, B. Mennucci, H. P. Hratchian, J. V Ortiz, A. F. Izmaylov, J. L. Sonnenberg, D. Williams-Yung, F. Ding, F. Lipparini, F. Egidi, J. Goings, B. Peng, A. Petrone, T. Henderson, D. Ranasinghe, V. G. Zakrzewski, J. Gao, N. Rega, G. Zheng, W. Liang, M. Hada, M. Ehara, K. Toyota, R. Fukuda, J. Hasegawa, M. Ishida, T. Nakajima, Y. Honda, O. Kitao, H. Nakai, T.

Vreven, K. Throssell, J. A. Montgomery Jr., J. E. Peralta, F. Ogliaro, M. Bearpark, J. J. Heyd, E. Brothers, K. N. Kudin, V. N. Staroverov, T. A. Keith, R. Kobayashi, J. Normand, K. Raghavachari, A. Rendell, J. C. Burant, S. S. Iyengar, J. Tomasi, M. Cossi, J. M. Millam, M. Klene, C. Adamo, R. Cammi, J. W. Ochterski, R. L. Martin, K. Morokuma, O. Farkas, J. B. Foresman and D. J. Fox, 2016.

18 C. Angeli, R. Cimiraglia, S. Evangelisti, T. Leininger and J.-P. Malrieu, Introduction of n-electron valence states for multireference perturbation theory, *J. Chem. Phys.*, 2001, **114**, 10252–10264.

19 C. Angeli, R. Cimiraglia and J.-P. Malrieu, n-electron valence state perturbation theory: A spinless formulation and an efficient implementation of the strongly contracted and of the partially contracted variants, *J. Chem. Phys.*, 2002, **117**, 9138–9153.

20 D. Schweinfurth, J. Krzystek, I. Schapiro, S. Demeshko, J. Klein, J. Telser, A. Ozarowski, C.-Y. Su, F. Meyer, M. Atanasov, F. Neese and B. Sarkar, Electronic Structures of Octahedral Ni(II) Complexes with "Click" Derived Triazole Ligands: A Combined Structural, Magnetometric, Spectroscopic, and Theoretical Study, *Inorg. Chem.*, 2013, **52**, 6880–6892.

21 J. Zheng, X. Xu and D. G. Truhlar, Minimally augmented Karlsruhe basis sets, *Theor. Chem. Acc.*, 2011, **128**, 295–305.

22 A. Filipponi, A. Di Cicco and C. R. Natoli, X-ray-absorption spectroscopy and n-body distribution functions in condensed matter. I. Theory, *Phys. Rev. B*, 1995, **52**, 15122–15134.

23 A. Filipponi and A. Di Cicco, X-ray-absorption spectroscopy and n-body distribution functions in condensed matter. II. Data analysis and applications, *Phys. Rev. B*, 1995, **52**, 15135–15149.

24 L. Hedin and S. Lundqvist, Effects of Electron-Electron and Electron-Phonon Interactions on the One-Electron States of Solids, *Solid State Phys.*, 1970, **23**, 1–181.

25 P. D'Angelo, V. Barone, G. Chillemi, N. Sanna, W. Meyer-Klaucke and N. V. Pavel, Hydrogen and higher shell contributions in Zn^{2+}, Ni^{2+}, and Co^{2+} aqueous solutions: An X-ray absorption fine structure and molecular dynamics study, *J. Am. Chem. Soc.*, 2002, **124**, 1958–1967.

26 M. Busato, A. Lapi, P. D'Angelo and A. Melchior, Coordination of the Co^{2+} and Ni^{2+} Ions in Tf_2N^- Based Ionic Liquids: A Combined X-ray Absorption and Molecular Dynamics Study, *J. Phys. Chem. B*, 2021, **125**, 6639–6648.

27 M. Benfatto, S. Della Longa and C. R. Natoli, The MXAN procedure: a new method for analysing the XANES spectra of metalloproteins to obtain structural quantitative information, *J. Synchrotron Radiat.*, 2003, **10**, 51–57.

28 T. A. Tyson, K. O. Hodgson, C. R. Natoli and M. Benfatto, General multiple-scattering scheme for the computation and interpretation of x-ray-absorption fine structure in atomic clusters with applications to SF_6, $GeCl_4$ and Br_2 molecules, *Phys. Rev. B*, 1992, **46**, 5997–6019.

29 J. E. Müller, O. Jepsen and J. W. Wilkins, X-ray absorption spectra: K-edges of 3d transition metals, L-edges of 3d and 4d metals, and M-edges of palladium, *Solid State Commun.*, 1982, **42**, 365–368.
